# Structural surfaceomics reveals an AML-specific conformation of integrin β_2_ as a CAR T cellular therapy target

**DOI:** 10.1038/s43018-023-00652-6

**Published:** 2023-10-30

**Authors:** Kamal Mandal, Gianina Wicaksono, Clinton Yu, Jarrett J. Adams, Michael R. Hoopmann, William C. Temple, Adila Izgutdina, Bonell Patiño Escobar, Maryna Gorelik, Christian H. Ihling, Matthew A. Nix, Akul Naik, William H. Xie, Juwita Hübner, Lisa A. Rollins, Sandy M. Reid, Emilio Ramos, Corynn Kasap, Veronica Steri, Juan Antonio Camara Serrano, Fernando Salangsang, Paul Phojanakong, Melanie McMillan, Victor Gavallos, Andrew D. Leavitt, Aaron C. Logan, Cliona M. Rooney, Justin Eyquem, Andrea Sinz, Benjamin J. Huang, Elliot Stieglitz, Catherine C. Smith, Robert L. Moritz, Sachdev S. Sidhu, Lan Huang, Arun P. Wiita

**Affiliations:** 1 Department of Laboratory Medicine, University of California San Francisco, San Francisco, CA USA; 2 Department of Physiology and Biophysics, University of California Irvine, Irvine, CA USA; 3 The Donnelly Centre, University of Toronto, Toronto, Ontario Canada; 4 School of Pharmacy, University of Waterloo, Kitchener, Ontario Canada; 5 Institute for Systems Biology, Seattle, WA USA; 6 Department of Pediatrics, Division of Hematology/Oncology, University of California San Francisco, San Francisco, CA USA; 7 Department of Pediatrics, Division of Allergy, Immunology, and Bone Marrow Transplantation, University of California San Francisco, San Francisco, CA USA; 8 Department of Pharmaceutical Chemistry and Bioanalytics, Institute of Pharmacy, Martin-Luther University Halle-Wittenberg, Halle, Germany; 9 UCSF/Gladstone Institute for Genomic Immunology, San Francisco, CA USA; 10 Helen Diller Family Comprehensive Cancer Center, University of California San Francisco, San Francisco, CA USA; 11 Center for Cell and Gene Therapy, Baylor College of Medicine, Houston Methodist Hospital–Texas Children’s Hospital, Houston, TX USA; 12 Department of Medicine, Division of Hematology/Oncology, University of California San Francisco, San Francisco, CA USA; 13 Department of Bioengineering and Therapeutic Sciences, University of California San Francisco, San Francisco, CA USA; 14 Chan Zuckerberg Biohub San Francisco, San Francisco, CA USA

**Keywords:** Cancer immunotherapy, Acute myeloid leukaemia, Proteomics, Cancer

## Abstract

Safely expanding indications for cellular therapies has been challenging given a lack of highly cancer-specific surface markers. Here we explore the hypothesis that tumor cells express cancer-specific surface protein conformations that are invisible to standard target discovery pipelines evaluating gene or protein expression, and these conformations can be identified and immunotherapeutically targeted. We term this strategy integrating cross-linking mass spectrometry with glycoprotein surface capture ‘structural surfaceomics’. As a proof of principle, we apply this technology to acute myeloid leukemia (AML), a hematologic malignancy with dismal outcomes and no known optimal immunotherapy target. We identify the activated conformation of integrin β_2_ as a structurally defined, widely expressed AML-specific target. We develop and characterize recombinant antibodies to this protein conformation and show that chimeric antigen receptor T cells eliminate AML cells and patient-derived xenografts without notable toxicity toward normal hematopoietic cells. Our findings validate an AML conformation-specific target antigen and demonstrate a tool kit for applying these strategies more broadly.

## Abstract (web summary)

Wiita and colleagues use cross-linking mass spectrometry and glycoprotein surface capture to identify an activated conformation of integrin β_2_ present on AML cells and develop CAR T cells that target this conformation antigen in preclinical models.

## Main

Cellular therapies are one of the most exciting modalities in cancer care^1^. However, safely applying these therapies to cancers beyond B cell malignancies has remained clinically challenging^2^. A major hurdle remains in the identification of surface antigens that are specifically expressed on tumor cells but not on other essential tissues, with the goal of minimizing ‘on target, off tumor’ toxicity^3,4^.

Recently, we were intrigued by the discovery of an activated conformation of integrin β_7_ as a specific cellular therapy in multiple myeloma^5^. This change in protein state led to the opportunity to target the active conformation of integrin β_7_ while sparing other normal blood cells, where this protein remained in the closed resting conformation. This finding raised the exciting hypothesis that given aberrancies in tumor signaling, metabolism or cell–microenvironment communication, cancer-specific surface protein conformations may in fact be widespread. However, this result in myeloma was the serendipitous outcome of a hybridoma screen. Thus, here, we aimed to develop a technology to systematically probe this possible untapped source of tumor-specific surface antigens. Specifically, we took advantage of cross-linking mass spectrometry (XL–MS)^6^. Although XL–MS is most often used to define protein–protein interactions or structural constraints^6^, this approach can also yield low-resolution structural information for hundreds or thousands of proteins in a sample^7,8^.

However, a major hurdle in XL–MS is the low fraction of cross-linked peptides compared to total peptides in any given sample^9^. Therefore, to focus on cell surface antigens, we combined XL–MS with cell surface capture (CSC), a method to specifically enrich cell surface N-linked glycoproteins^10^. We and others have used CSC to successfully identify immunotherapy targets based on surface protein abundance^11,12^. Here, by combining XL–MS and CSC in ‘structural surfaceomics’, we aim to move to the next level of protein-centric target discovery.

As an initial proof of principle, we apply structural surfaceomics to acute myeloid leukemia (AML), a frequently diagnosed hematologic malignancy with dismal prognosis^13^. Thus far, chimeric antigen receptor (CAR) T cells in AML have generally led to either notable toxicities or disappointing clinical efficacy^14,15^. One major hurdle to CAR T therapy for AML is lack of optimal immunotherapy targets^12^. Leading targets include CD33, CD123 and CLL-1/CLEC12A. However, these are all expressed not only on AML blasts but also on normal myeloid cells and/or hematopoietic stem and progenitor cells (HSPCs), driving the potential for major toxicity as well as issues of intratumoral heterogeneity, leading to suboptimal efficacy^12,14,16,17^. Thus, there remains a great need to identify AML-specific cellular therapy targets that may eliminate tumor cells while sparing normal myeloid cells.

Here, we apply structural surfaceomics to an AML model and identify the activated conformation of integrin β_2_ as a promising immunotherapeutic target. We develop and characterize humanized recombinant antibodies specific to the activated conformation of this protein. We further demonstrate that CAR T cells that target integrin β_2_ are efficacious in AML models and, importantly, do not show any evidence of toxicity in normal hematopoietic cells in a humanized immune system (HIS) mouse model, unlike anti-CD33 CAR T cells. In addition, our findings suggest structural surfaceomics as a strategy to unlock a previously unexplored class of immunotherapy targets invisible to standard discovery strategies.

## Results

### Development of the structural surfaceomics technology

**Fig. 1:**
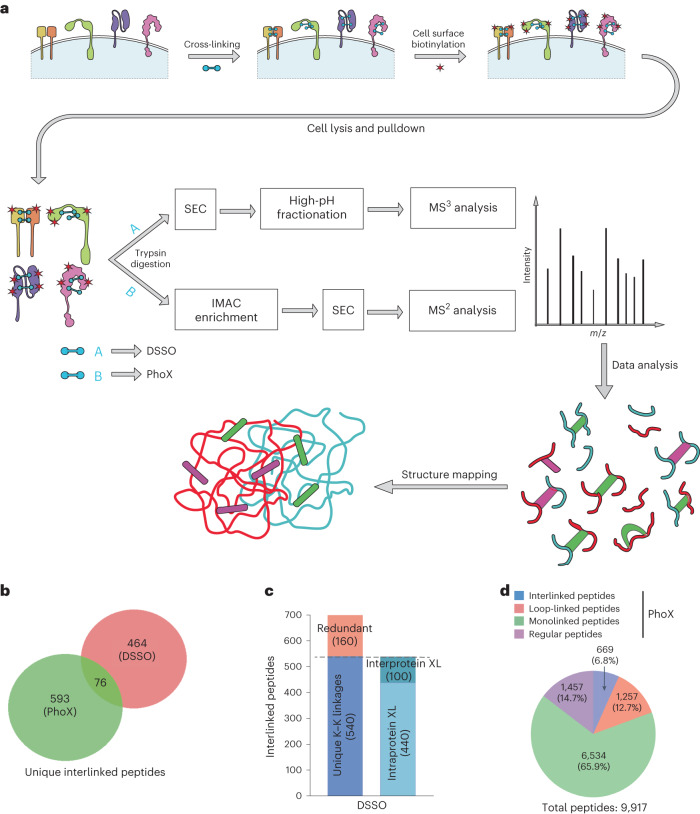
XL–MS and surface glycoprotein capture strategy to identify conformation-specific cancer antigens. **a**, Schematic flow diagram of the ‘structural surfaceomics’ approach. **b**, Venn diagram showing the total number of cross-linked peptides identified from the two different approaches (tandem MS (MS^2^) and multistage MS (MS^3^) based). PhoX and DSSO were used as cross-linkers for the MS^2^ and MS^3^ approaches, respectively. **c**, Bar graph showing the distribution of inter- and intraprotein cross-links (XL) from MS^3^-based (DSSO) XL–MS. **d**, Pie chart showing the distribution of the various types of cross-links obtained from PhoX MS^2^-based XL–MS. All cross-links were identified with a ≤1% FDR (see Methods for details). ‘Regular’ peptides indicate that no PhoX modification was detected on any lysines.

Our overall strategy for structural surfaceomics is to first use a bifunctional chemical cross-linker applied to live cells, followed by glycoprotein oxidation and biotinylation using the CSC strategy (Fig. 1a). Our goal is to ‘freeze’ the native protein conformation in situ, thereby preserving relevant structural information, and use streptavidin-based enrichment of surface proteins to increase MS coverage of our most relevant peptides.

As an initial model system, we used the Nomo1 AML cell line, which is derived from an individual with monocytic leukemia^18^. Using Nomo1 cells, we explored two complementary XL–MS strategies. One strategy incorporates the MS-cleavable cross-linker disuccinimidyl sulfoxide (DSSO), which we and others have used frequently to study protein–protein interactions^19–21^. We also used the recently described non-cleavable cross-linker disuccinimidyl phenyl phosphonic acid (PhoX), which incorporates a phosphonate-based handle for enrichment via immobilized metal affinity chromatography (IMAC)^9^. We applied these strategies in separate experiments to Nomo1 cells using cellular input of 0.4 × 10^9^–5 × 10^9^ cells (Fig. 1a,b).

**Extended Data Fig. 1:**
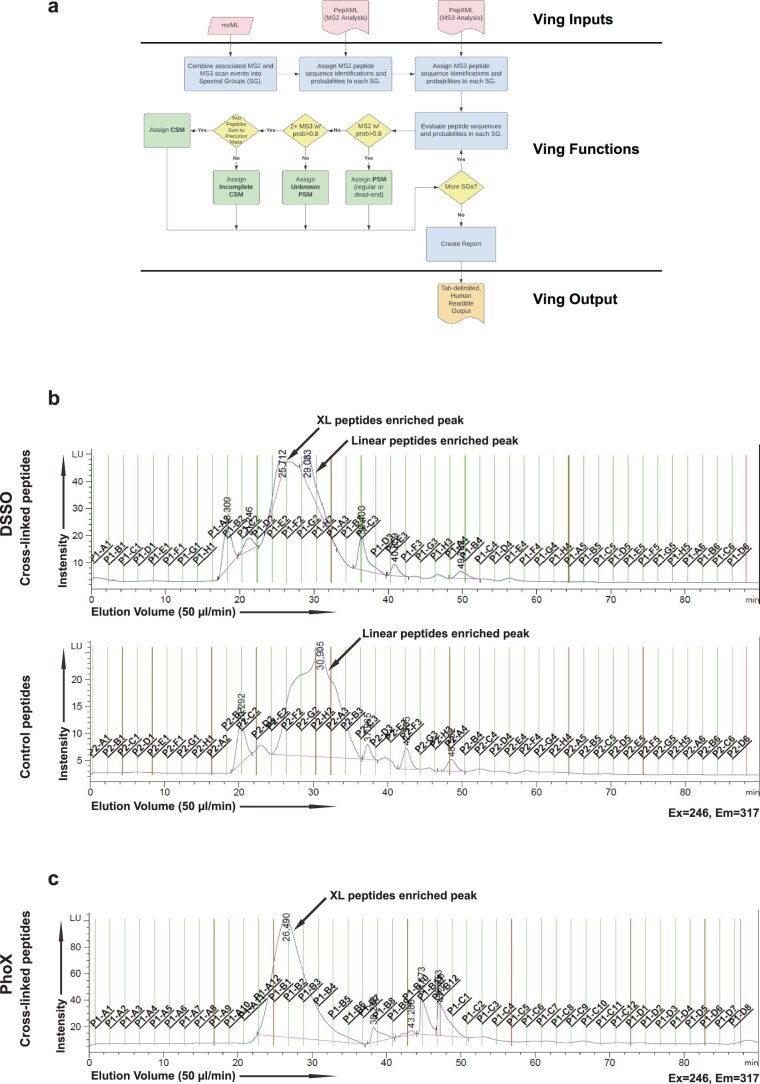
Ving and XL-MS SEC. **a**, Schematic workflow describing the working principle of Ving. **b**, Representative SEC trace of peptides obtained from DSSO cross-linked samples. **c**, Representative SEC trace of peptides obtained from PhoX cross-linked samples. For both strategies (DSSO and PhoX), samples were processed in 4 separate batches and each time the SEC trace pattern was similar. XL peptides refers to cross-linked peptides.

**Extended Data Fig. 2:**
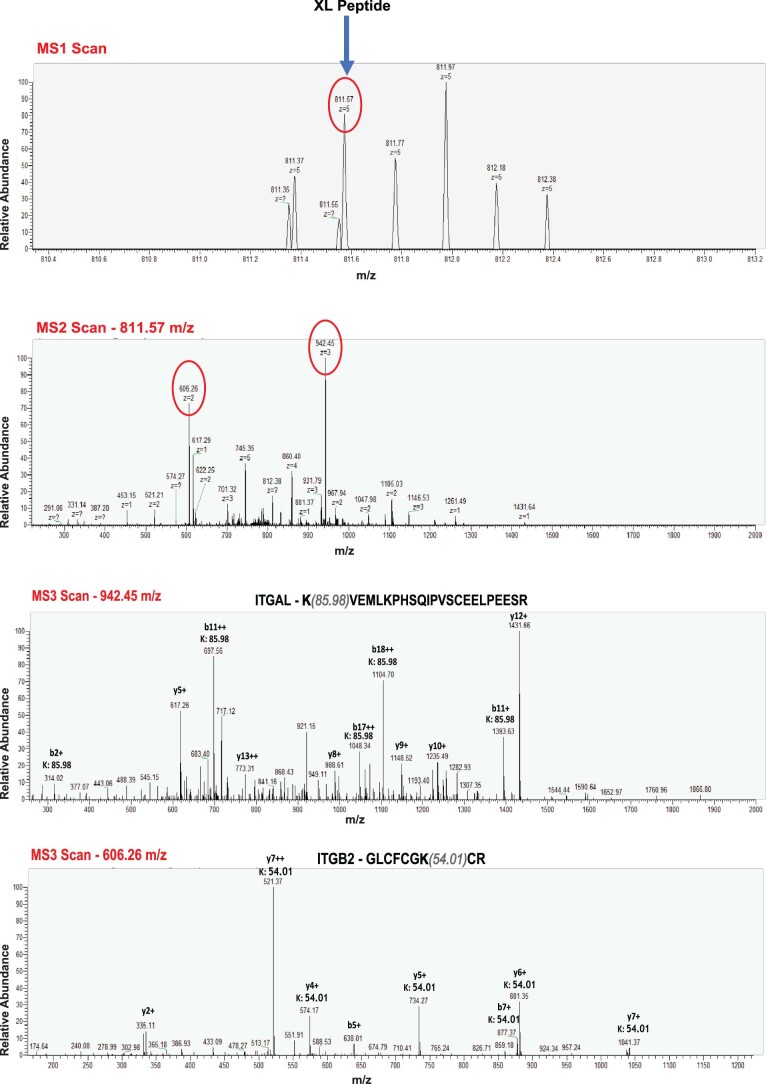
Representative MS spectra demonstrating the MS3 based strategy of XL-MS. Example MS spectra demonstrating the MS^3^ based strategy of XL-MS. The cross-linked peptides of 811.57 *m*/*z* is selected for MS^2^, at which step the cross-linker is cleaved in the collision cell generating two separate peptides of 606.26 *m*/*z* and 942.45 *m*/*z*, respectively. These two high-abundance peptides are then selected for MS^3^ where they undergoes full fragmentation for peptide identification. We also note the respective modification on Lysine residues resulting from the cross-linker.

XL–MS can identify interlinked (type 2; bridging two separate peptides), intralinked (‘loop linked’, type 1; two lysines cross-linked in the same peptide) and monolinked (‘dead end’, type 0; single modified lysine) peptides. Inter- and intralinked peptides could be informative for our strategy, whereas monolinked peptides are not. For DSSO, we used our previously published computational approach^19^ to analyze these data and also adapted this strategy to a publicly available version compatible with the Trans-Proteomic Pipeline (TPP)^22^, called Ving (Extended Data Figs. 1a and 2 and Methods). In our initial DSSO experiment, we enriched cross-linked peptides by size-exclusion chromatography (SEC) alone, whereas in our subsequent experiment, we followed SEC with tip-based, reversed-phase high-pH fractionation (HpHt) to optimize coverage^23^. Between these two DSSO experiments, a total of 700 unique interlinked peptides from 236 proteins were identified (Fig. 1c). Of these cross-links, 42.4% mapped to UniProt-annotated membrane-spanning proteins, demonstrating a strong focus on this compartment. The PhoX sample, processed using IMAC and SEC, resulted in 85.3% of total peptides demonstrating a cross-linked lysine (669 unique interlinks, 1,257 loop links and 6,534 uninformative monolinks), derived from 782 proteins (Fig. 1d). Although enrichment for membrane-spanning proteins for PhoX was less than DSSO, at 27.9%, this value was still broadly consistent with our prior studies using CSC alone^24^. Combining these data, our ‘structural surfaceomics’ approach identified 2,390 total interlinked and intralinked peptides on Nomo1 cells.

### Active conformation of integrin β_2_ as a potential AML target

**Fig. 2:**
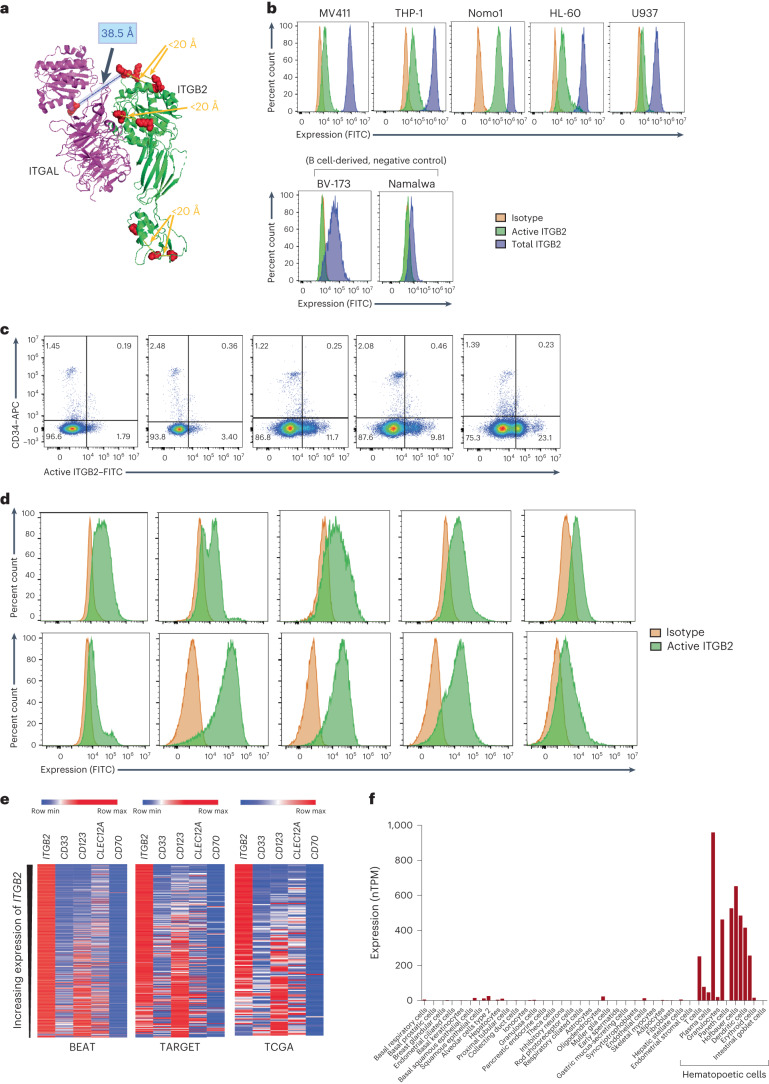
Activated integrin β_2_ is a conformationally selective antigen in AML. **a**, Identified cross-linked peptides mapped on to the crystal structure of the integrin α_L_/integrin β_2_ heterodimer (PDB 5E6R). **b**, Flow cytometry histogram plot showing expression of total and activated integrin β_2_ on AML (top) and B cell lines (bottom; BV-173 and Namalwa). The *y* axis represents percent count normalized to mode. The gating strategy is shown in Supplementary Information 1a. Data are representative of *n* = 4 (Nomo1), 2 (THP-1) and 1 (all others) independent experiments. **c**, Flow cytometry plot showing the absence of active integrin β_2_ on CD34^+^ HSPCs from GM-CSF-mobilized peripheral blood. The gating strategy is shown in Supplementary Information 1b. Deidentified human samples were used for this analysis (*n* = 5 independent donors). **d**, Representative flow cytometry histogram plots showing the expression of active integrin β_2_ on primary AML cells. The *y* axis represents percent count normalized to mode. The gating strategy is shown in Supplementary Information 1c. Data are representative of *n* = 10 total deidentified samples. **e**, Heat map showing inverse expression patterns of *ITGB2* against other AML targets in publicly available primary AML RNA-seq data. The color bar represents maximum expression in each row based on normalized read counts. The sample sizes of BEAT^37^ AML (adult), TARGET^38^ (pediatric) and TCGA^36^ were 510, 255 and 150, respectively. **f**, Aggregated single-cell RNA-seq data showing essentially exclusive expression of *ITGB2* in hematopoietic tissue; data were obtained from the Human Protein Atlas^39^; nTPM, normalized transcripts per million.

**Extended Data Fig. 3:**
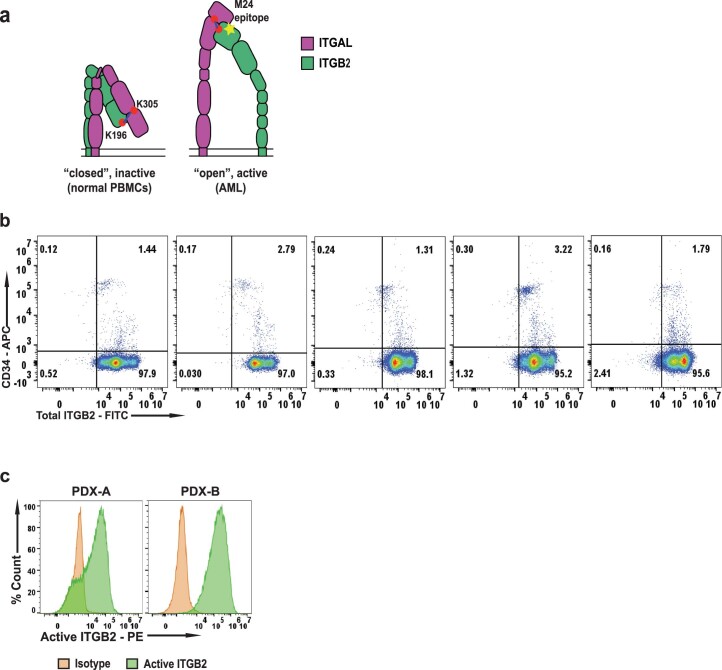
Discerning active integrin β_2_ expression. **a**, Cartoon diagram showing proposed inactive and active conformations of ITGB2. **b**, Flow cytometry plot showing presence of total integrin β_2_ on CD34+ HSPCs from GM-CSF mobilized peripheral blood. Cells were gated on singlet cells for analysis. Deidentified patient samples were used for this analysis (*n* = 5, independent donors). **c**, Flow cytometry analysis showing expression of active ITGB2 in PDX models of AML (PDX-A and PDX-B). The *y*-axis represents percent count normalized to mode. Cells were gated on human CD45+ population cells for analysis. Representative plot from *n* = 2 separate PDX models of AML.

We manually compared the cross-linked peptides obtained from structural surfaceomics to published structures in the Protein Data Bank (PDB). In DSSO data, we were particularly intrigued to find several cross-links mapping to the protein integrin β_2_ and its heterodimer partner integrin α_L_ (PDB 5E6R)^25^. We first noted several intraprotein cross-links within integrin β_2_ itself that fit the Cα lysine–lysine distance constraints of the DSSO cross-linker, <20 Å. However, we found four cross-links that did not match the Cα–Cα distance constraint on the available crystal structure, extending to ~38.5 Å between Lys 194 and Lys 196 of the βI domain of integrin β_2_ and Lys 305 and Lys 330 on the I domain of integrin α_L_^25,26^ (Fig. 2a). Notably, the crystal structure appears to represent the inactive, closed form of this integrin heterodimer^25,26^. Our XL–MS data suggested that these domains are instead in closer proximity on Nomo1 cells, potentially consistent with the open, active conformation in these AML tumor cells (Extended Data Fig. 3a).

This finding was notable as integrin β_2_ has been identified on several immune cell types, including monocytes, neutrophils, natural killer cells and T cells^27,28^. However, at the protein level, it is known to largely remain in the closed, resting conformation until cellular activation after exposure to appropriate cytokines, adhesion molecules or other proteins^29–32^. Furthermore, a previous study suggested that constitutive signaling through integrin β_2_ maintains proliferation in AML blasts^33^. Taken together, these results suggest that aberrant AML biology may lead to constitutive activation of integrin β_2_, thus creating a possible tumor-specific conformation that, when targeted, would largely spare normal resting hematopoietic cells.

To explore this hypothesis, we took advantage of the mouse monoclonal antibody M24, which is widely used to selectively recognize the activated form of integrin β_2_ by flow cytometry^34^. We confirmed that four AML cell lines of varying genotypes (Nomo1, THP-1, HL-60 and MV-4-11) all showed clear M24 staining, in addition to high levels of total integrin β_2_ as detected by the TS1/18 clone (Fig. 2b). By contrast, the B cell malignancy cell lines BV-173 and Namalwa showed total integrin β_2_ expression but no discernable expression of the activated conformation (Fig. 2b). To extend this result to normal hematopoietic progenitors, we further obtained granulocyte–macrophage colony-stimulating factor (GM-CSF)-mobilized peripheral blood samples from five hematopoietic stem cell transplant donors at our institution. We found that CD34^+^ HSPCs from these individuals showed no evidence of activated integrin β_2_ by flow cytometry (Fig. 2c), although they did express total integrin β_2_ (Extended Data Fig. 3b). This result provides an initial suggestion of a favorable therapeutic index for this target.

**Extended Data Fig. 4:**
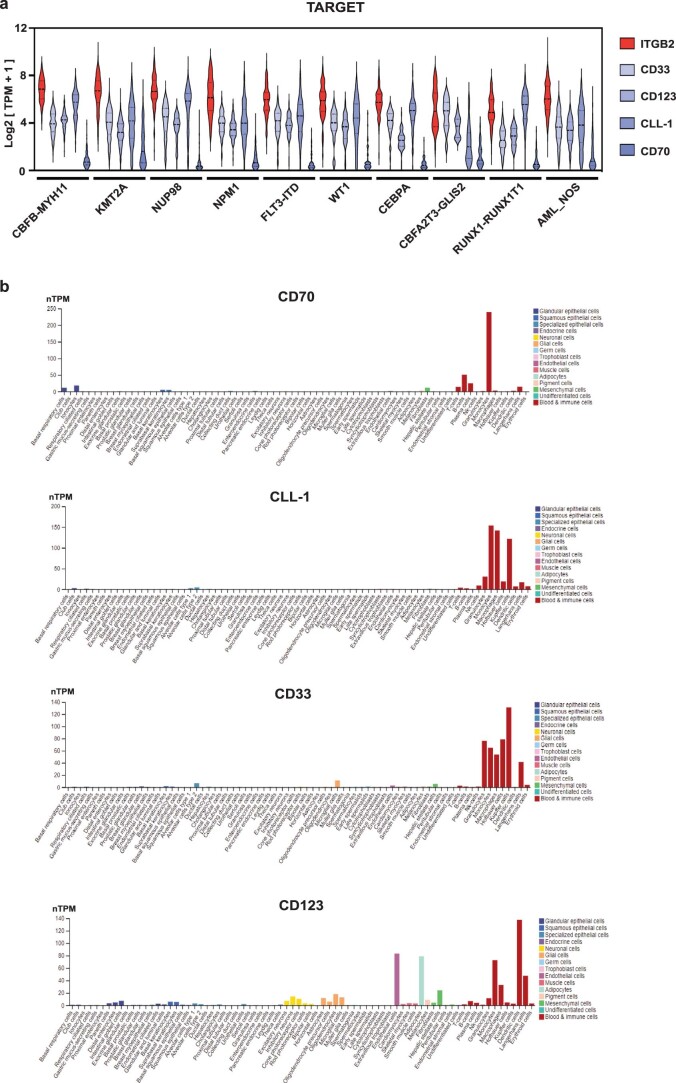
ITGB2 transcript expression. **a**, AML subtype specific expression analysis of *ITGB2* and other notable AML targets of patient samples from TARGET database. **b**, Single cell sequencing data showing expression of notable AML target across normal human tissues and immune cells (adapted from Human Protein Atlas^42^).

To further evaluate activated integrin β_2_ in primary AML, we obtained deidentified bone marrow aspirate specimens from ten individuals at our institution (Fig. 2d) and two patient-derived xenograft (PDX) models of AML from the Public Repository for Xenografts (PRoXe) biobank^35^ (Extended Data Fig. 3c). Gating on mature blasts, we found that activated integrin β_2_ appeared highly expressed in 9 of 12 total samples analyzed. We further analyzed bulk RNA-sequencing (RNA-seq) data across three AML tumor datasets (The Cancer Genome Atlas (TCGA) and BEAT AML datasets (adult) and the TARGET dataset (pediatric))^36–38^ and found high blast expression of *ITGB2* transcript across AML genotypes as well as potential complementarity with *CD33* and *IL3RA* (CD123) (Fig. 2e and Extended Data Fig. 4a). However, we note that transcript expression alone cannot report whether surface integrin β_2_ is in the activated or resting conformation. Toward the safety profile of this target, we evaluated single-cell RNA-seq data in the Human Protein Atlas^39^. *ITGB2* transcript was only detectably expressed in hematopoietic cell types (Fig. 2f), with high expression across the myeloid lineage^31,32^. Already, this transcript expression pattern compares favorably with that of other known AML immunotherapy targets (Extended Data Fig. 4b). However, we anticipate that conformation-selective targeting will lead to an additional layer of discrimination between tumor and normal cells not available to other targets.

### Characterization of antibody binders for active integrin β_2_

**Extended Data Fig. 5:**
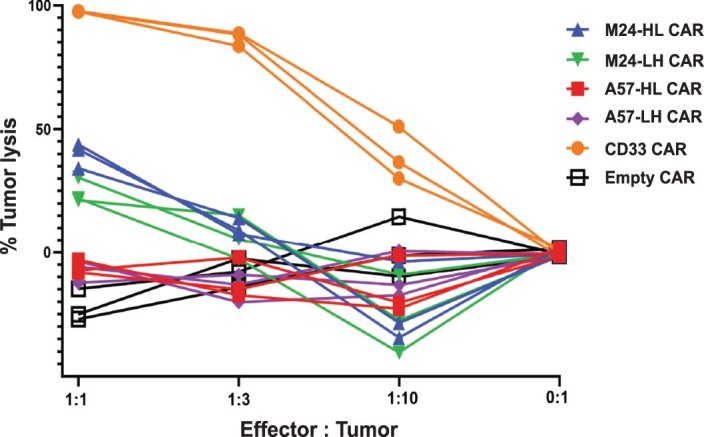
Initial anti-active integrin β_2_ CAR-T designs. Luciferase based cytotoxicity analysis of M24 and A57 antibody derived CAR-T cells against AML cell line Nomo-1 (Data from single experiment performed in triplicate). The luciferase signals of the cytotoxicity assays were normalized against untransduced CAR-T of their respective E:T ratios.

We next sought to develop CAR T cells targeted to active integrin β_2_ as a proof-of-principle therapeutic for AML. We first explored two commercially available antibody clones to active integrin β_2_, M24 (ref. ^40^) and AL57 (ref. ^41^). Using the sequence of these antibodies, we designed single-chain variable fragment (scFv) binders and incorporated them into a CD28-based CAR backbone. Although we found no activity for AL57-based scFvs, we did find that both the designs (Vh–Vl and Vl–Vh) of the M24-derived scFv did indeed lead to some Nomo1 cytotoxicity (Extended Data Fig. 5). Here and throughout the study, we also used a previously described anti-CD33 CAR as a positive control^16^.

Although this result was promising that CAR T cells targeting integrin β_2_ could be developed, these M24-derived CAR T cells showed relatively limited in vitro potency. Furthermore, the M24 framework sequences are fully murine^40^, increasing the potential for immunogenicity in humans. Therefore, we sought to develop alternative CAR T cell designs.

**Fig. 3:**
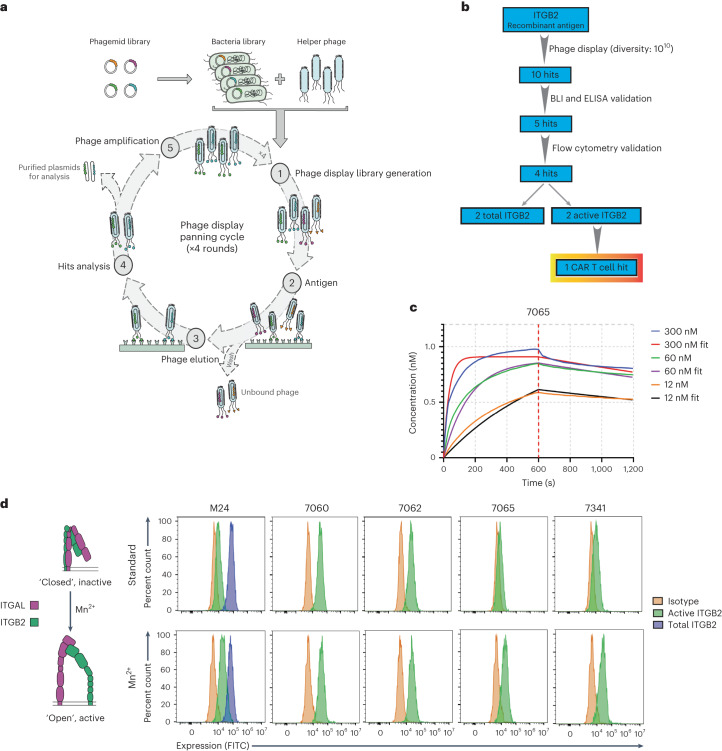
Antibody 7065 binds preferentially to the active conformation of integrin β_2_. **a**, Schematic flow diagram of the phage display selection strategy used for developing antibodies to integrin β_2_. **b**, Schematic flow diagram showing triage of antibodies obtained from the phage display library and the downstream validation/funneling to identify an active integrin β_2_ binder. **c**, Representative BLI plot showing determination of binding affinity (*K*_d_) of the 7065 antibody to integrin α_L_/integrin β_2_; *n* = 3 different concentrations of antibody were used for this experiment (see also Extended Data Fig. 6c). **d**, Flow cytometry analysis of Jurkat T-ALL cells in the presence and absence of 2 mM Mn^2+^ ions to determine/identify antibodies with specificity to active integrin β_2_. The *y* axis represents percent count normalized to mode. The gating strategy is shown in Supplementary Information 1a. Data are representative of *n* = 2 independent experiments.

**Extended Data Fig. 6:**
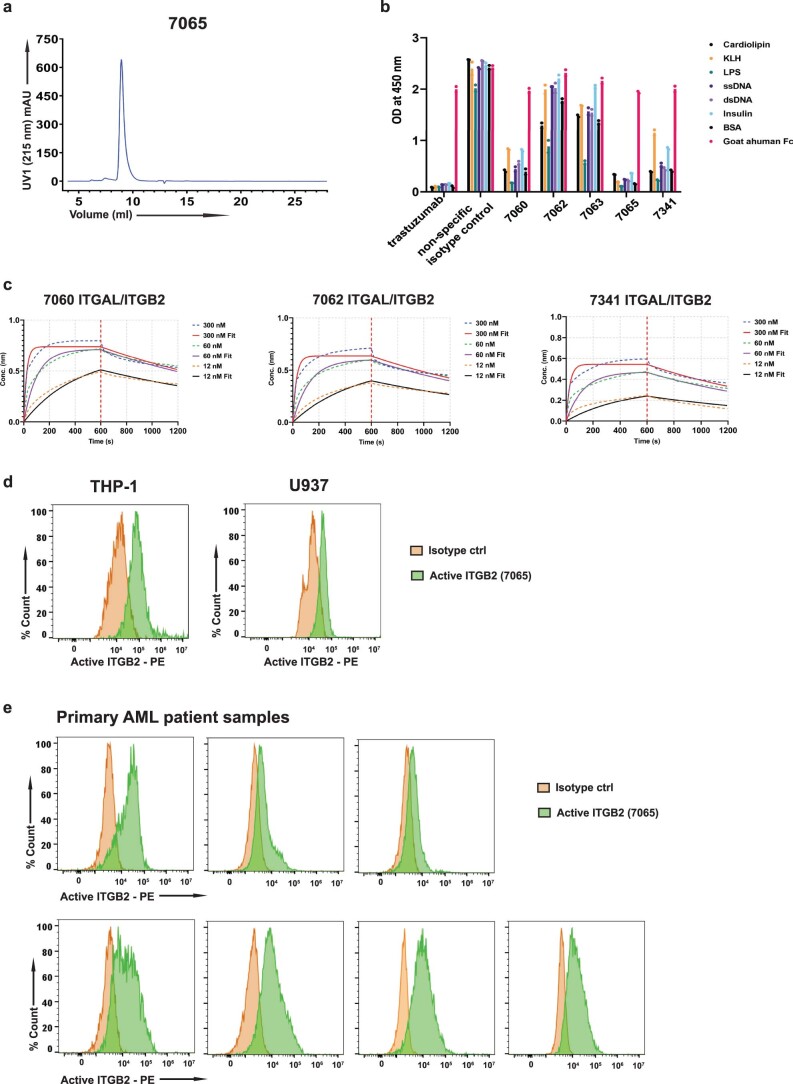
Characterizing recombinant antibodies to integrin β_2_. **a**, Representative SEC trace of the antibody 7065 showing distinct peak, for quality check. **b**. Non-specific ELISA panel showing specificity profiles of the antibodies obtained from phage display selection. (Data from single experiment performed in duplicates) **c**, Representative BLI plots showing binding affinities (K_D_) of the antibodies against ITGB2 with their alpha partners. Each experiment was performed with *n* = 3 different concentrations of antibody. **d**, Flow cytometry analysis showing binding of antibody 7065 on the AML cells. The y-axis represents percent count normalized to mode. Gating strategy shown in Supplementary Information 1a. *n* = 3 independent experiments. **e**, Flow cytometry analysis showing binding of antibody 7065 on the primary AML cells, gated on CD34+ blasts. The y-axis represents percent count normalized to mode. Gating strategy shown in Supplementary Information 1c. *n* = 7 independent patient samples.

As a first step, we used our previously described antigen-binding fragment (Fab) phage display platform^42^ based on a fully human framework sequence to perform selections versus recombinant integrin β_2_ (Fig. 3a). From a library of ~10^10^ binders, we identified ten initial hits versus integrin β_2_, five of which were validated by biolayer interferometry (BLI) and nonspecific enzyme-linked immunosorbent assays (ELISAs; Fig. 3b,c and Extended Data Fig. 6a–c) to have binding affinities to integrin β_2_ in the low-nanomolar range and to lack binding of irrelevant proteins, respectively (Extended Data Fig. 6b,c and Supplementary Table 1). These five Fabs were cloned into a human IgG1 backbone and recombinantly expressed in mammalian cells (Extended Data Fig. 6a). As a validation system, we chose Jurkat T-ALL cells, which we found express high levels of integrin β_2_ with a fraction appearing to show constitutive activation at baseline (Fig. 3d). Encouragingly, four of our five recombinant antibodies to integrin β_2_ showed positive signal by flow cytometry (Fig. 3d).

We next took advantage of the fact that integrins can be biochemically converted from the inactive, closed conformation to the active, open conformation by treatment with the divalent cation Mn^2+^ (ref. ^43^). Although two clones (7060 and 7062) did not show any responsiveness to 2 mM Mn^2+^ treatment, clones 7065 and 7341 showed increased signal in response to Mn^2+^ (Fig. 3d). Indeed, the profile of 7065 appeared similar to the well-validated antibody M24, with limited signal in the absence of Mn^2+^ but an approximately threefold increase in mean fluorescence intensity after cation exposure. The higher signal from 7341 at baseline suggests that it may also have some binding to the closed conformation of integrin β_2_. Flow cytometry of primary AML samples and cell lines with clone 7065 (Extended Data Fig. 6d,e) showed a similar profile as those stained with M24 (Fig. 2d). These findings suggest that clone 7065 may be particularly selective for the activated conformation of integrin β_2_.

### Development of antiactive integrin β_2_ CAR T cells

**Fig. 4:**
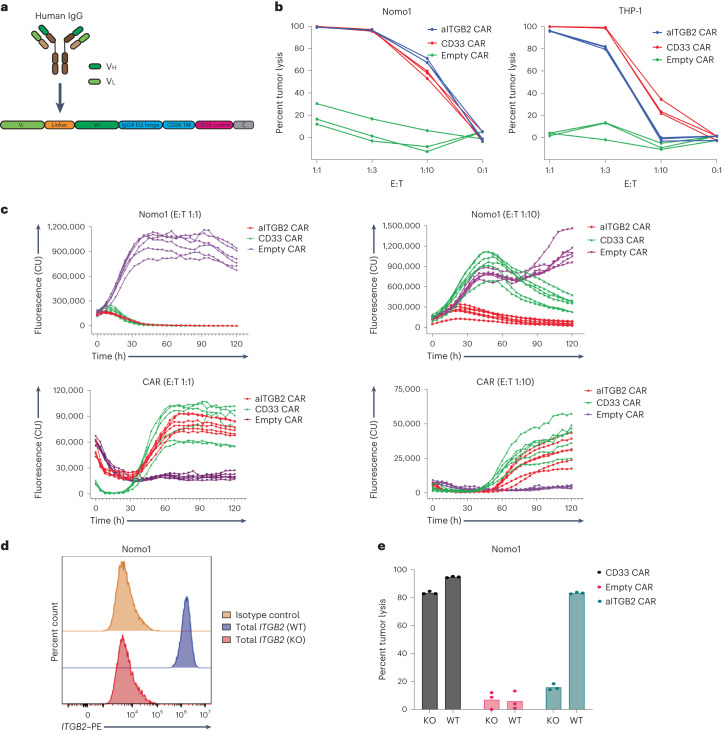
aITGB2 CAR T cells derived from the 7065 antibody are cytotoxic to AML cells. **a**, Schematic diagram of the CAR T construct used; TM, transmembrane; costim, co-stimulatory domain. **b**, Luciferase-based cytotoxicity of the aITGB2 CAR T design in Nomo1 and THP-1 AML cell lines. Data are representative of *n* = 3 independent experiments with similar results. Each experiment was performed in triplicate. **c**, Incucyte live-cell imaging data demonstrating efficient cytotoxicity of aITGB2 CAR T cells against Nomo1 cells at two different E:T ratios (1:1 and 1:10) over a 5-d period. CAR T cells were labeled with GFP, and tumor cells (Nomo1) were labeled with mCherry to facilitate fluorescence-based quantification. The *y* axis represents integrated fluorescence used as a proxy to monitor cell proliferation. Data are from a single experiment performed with six replicates. CU, calibrated units. **d**, Flow cytometry histogram showing a successfully generated *ITGB2*-knockout version of Nomo1 cells using CRISPR–Cas9. The *y* axis represents percent count normalized to mode. The gating strategy is shown in Supplementary Information 1a; KO, knockout; WT, wild-type. **e**, Luciferase-based cytotoxicity data showing specific activity of aITGB2 CAR T cells against wild-type Nomo1 cells and not against *ITGB2*-knockout Nomo1 cells (the E:T ratio was 1:1 with overnight incubation). Data are representative of *n* = 2 independent experiments with similar results. Each experiment was performed in triplicate. The luciferase signals of the cytotoxicity assays were normalized against untransduced CAR T cells of their respective E:T ratios. Only aITGB2 CAR T cell manufacturing involved knocking out *ITGB2*.

**Extended Data Fig. 7:**
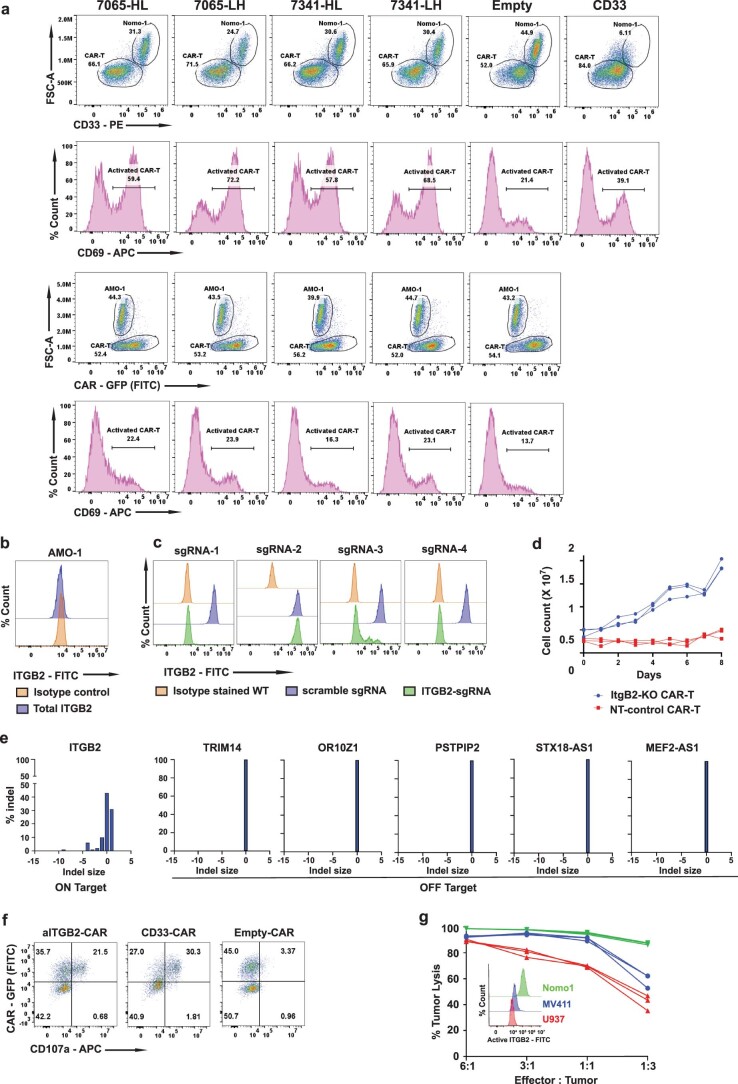
Evaluation of aITGB2 CAR-T designs incorporating recombinant antibodies. **a**, Flow cytometry screen for cytotoxicity and activation status of aITGB2 CAR-T designs vs. AML cell line Nomo1 (*n* = 1 for each design). Similarly, as a demonstration of specificity, cytotoxicity and activation status was also checked against AMO-1 (multiple myeloma cell line that does not express integrin β_2_). Cells were gated on single cells for analysis. Gating strategy shown in Supplementary Information 2a. **b**, Flow cytometry analysis showing absence of ITGB2 in AMO-1. Cells were gated on single cells for analysis. Flow cytometry gating strategy similar to shown in Supplementary Information 1a. **c**, Flow cytometry analysis showing knockout efficiency of the various sgRNA used for knocking out *ITGB2* in primary T cells (*n* = 1 for each sgRNA). Cells were gated on single cells for analysis. Flow cytometry gating strategy similar to that shown in Supplementary Information 1a. **d**, Plot showing proliferation of aITGB2-CAR-T cells, ‘with ITGB2 knockout’ vs ‘Non-Targeting (NT) – control’. Scrambled sgRNA was used for NT-control. (Data from single experiment performed in triplicate). **e**, Plots showing off-target analysis of the sgRNA used for CAR-T manufacturing using CRISPOR tool. Top 5 potential hits were assessed. **f**, Degranulation assay of aITGB2 and anti-CD33 CAR-T against Nomo1 based on CD107a staining. CAR positivity denoted by GFP tag on CAR construct. E:T ratio was 1:1 and 6 hours incubation time (*n* = 1). Cells were gated on single cells for analysis. Gating strategy similar to shown in Supplementary Information 2a. **g**, Luciferase assay-based cytotoxicity analysis showing cytotoxicity of aITGB2-CAR-T cells against AML cell lines with varying antigen density (Representative of *n* = 2 independent experiments with similar results, each experiment performed in triplicate). The flow cytometry data showing antigen density has been repurposed from Fig. 2b. Only aITGB2 CAR-T manufacturing involved knocking out integrin β_2_, and only in (f) and (g).

The sequences of 7065 and 7341 were next engineered into the scFv format and cloned into a CAR backbone with a CD28 co-stimulatory domain (Fig. 4a). For each antibody, we explored two different scFv orientations, either Vh–Vl or Vl–Vh, with a 3× Gly_4_-Ser linker. Based on Nomo1 cytotoxicity in vitro, the 7065 Vl–Vh design appeared to be more efficacious (Extended Data Fig. 7a) than control ‘empty’ CAR T cells (CAR backbone but no antibody binder). This 7065 design also showed no discernible activity versus the negative-control AMO-1 cell line, a multiple myeloma cell line that does not express activated integrin β_2_ (Extended Data Fig. 7a,b).

Although these initial in vitro experiments were promising, we did anecdotally observe decreased proliferation and final yield of these CAR T cells during manufacturing. Furthermore, even the best-performing CAR T cell design had moderate Nomo1 cytotoxicity compared to positive-control anti-CD33 CAR T cells (Extended Data Fig. 7a). We hypothesized that T cell stimulation was leading to integrin β_2_ activation, and thus some degree of CAR T cell ‘fratricide’, during expansion. To test this hypothesis, we used an approach used for other CAR T cell targets present on activated T cells, such as CD70 (ref. ^44^), where CRISPR–Cas9 ribonucleoprotein (RNP) is used to knock out *ITGB2* before T cell stimulation (Extended Data Fig. 7c). Single guide RNA 1 (‘sgRNA-1’) showed no evidence of genomic DNA cleavage at predicted off-target sites (Extended Data Fig. 7e) and was thus used for downstream manufacturing. Using this protocol, we no longer observed any deficit in CAR T cell expansion (Extended Data Fig. 7d), and, furthermore, we observed in vitro cytotoxicity versus Nomo1 and THP-1 cells comparable to anti-CD33 (Fig. 4b). The CAR T cells were also found to have potent degranulation against Nomo1 cells (Extended Data Fig. 7f). As expected, antitumor cytotoxicity correlated with antigen density (Fig. 2b and Extended Data Fig. 7g).

**Extended Data Fig. 8:**
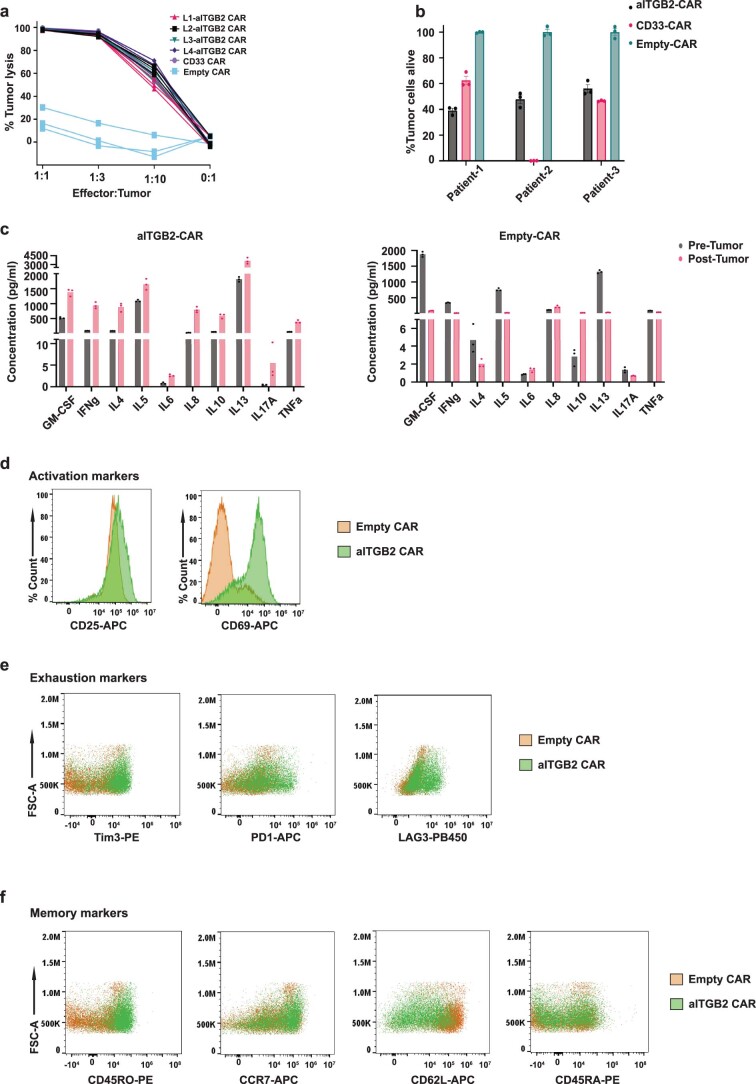
Additional aITGB2 CAR-T evaluation. **a**, Luciferase assay-based cytotoxicity analysis showing efficacy of 7065 based aITGB2 CAR-T with 1x – 4x Gly_4_Ser (L1-L4) linker between heavy and light chain (Data from single experiment performed in triplicate). The luciferase signals of the cytotoxicity assays were normalized against untransduced T-cells of their respective E:T ratios. **b**, Bar plots showing cytotoxicity of aITGB2 CAR-T against primary AML patient samples. *n* = 3 technical replicates and 3 independent patient samples. E:T ratio was 3:1 with overnight incubation. Cells were gated on CD34+ cells for analysis. Gating strategy similar to Supplementary Information 1c. **c**, Cytokine profiling of aITGB2 CAR-T comparing against Empty CAR-T control upon pre vs post tumor exposure at E:T ratio of 1:1 for overnight incubation (Data from single experiment performed in triplicate). **d**–**f**, Representative flow cytometry-based histogram analysis showing activation markers (**d**), exhaustion markers (**e**) and memory markers (**f**) of CAR-T in pre vs post tumor exposure at E:T ratio of 1:1 for overnight incubation. Gating strategy similar to Supplementary Information 2d. Antibody dilutions used in **d**–**f** was 1:50. Only aITGB2 CAR-T manufacturing involved knocking out integrin β_2_. All the statistical data in this figure are represented as mean ± SEM.

We further varied the Vl–Vh linker length and found largely consistent cytotoxicity (Extended Data Fig. 8a). We thus chose either the ‘3×’ or ‘4×’ linker designs as lead candidates for evaluation. To assess proliferation kinetics of antiactive integrin β_2_ (aITGB2) CAR T cells, we performed live-cell imaging assays of Nomo1 cocultures. We found that at a 1:1 effector-to-tumor (E:T) ratio, aITGB2 CAR T cells showed levels of proliferation and cytotoxicity that were similar to those observed with anti-CD33 CAR T cells (Fig. 4c). However, at a 1:10 E:T ratio, aITGB2 CAR T cells outperformed anti-CD33 (Fig. 4c). Both CAR T cells showed similar proliferation in this coculture assay (Fig. 4c). aITGB2 CAR T cells were also found to be efficacious against primary AML samples (Extended Data Fig. 8b) and led to increased release of several cytokines after tumor exposure compared to empty control CAR T cells (Extended Data Fig. 8c). Further characterization based on memory, exhaustion and activation signaling markers revealed favorable features of aITGB2 CAR T cells (Extended Data Fig. 8d–f). Taken together, these findings encourage preclinical investigation of aITGB2 CAR T cells as an AML therapy.

### aITGB2 CAR T cells are specific to active integrin β_2_

**Fig. 5:**
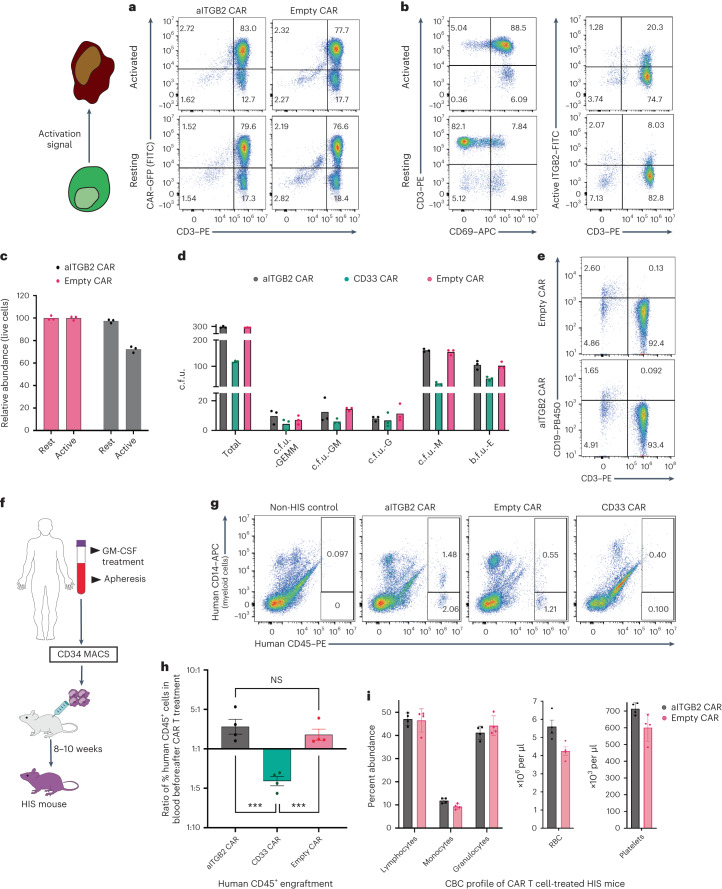
Toxicity assessment of aITGB2 CAR T cells demonstrates a promising safety profile. **a**, Flow cytometry-based cytotoxicity assay showing specificity of aITGB2 CAR T cells to activated peripheral blood T cells that harbor activated integrin β_2_ (lower right quadrant with CAR^–^ and CD3^+^ T cells). Both resting and activated conditions were performed in overnight coculture assays with aITGB2 CAR T cells. The gating strategy is similar to that shown in Supplementary Information 1b. **b**, Flow cytometry analysis showing successful activation of T cells and partial abundance of activated integrin β_2_ on activated T cells. The gating strategy is similar to that shown in Supplementary Information 1b. **c**, Quantitative analysis of active T cell depletion data in **a**. Data are representative of *n* = 2 independent experiments with similar results, one of which was performed in triplicate. **d**, Clonogenic assay showing no impact of aITGB2 CAR T cells against CD34^+^ HSPCs from GM-CSF-mobilized peripheral blood. The E:T ratio was 1:1 (see Methods section for details). Data are representative of *n* = 2 independent experiments with similar results. Each experiment was performed in triplicate; c.f.u., colony-forming units; GEMM, granulocyte, erythrocyte, monocyte, megakaryocyte; GM, granulocyte, monocyte; G, granulocyte; M, monocyte; E, erythrocyte; b.f.u., burst-forming units. **e**, Flow cytometry analysis showing no discernible impact of aITGB2 CAR T cells against T cells and B cells. The *y* axis represents percent count normalized to mode. The gating strategy is similar to that shown in Supplementary Information 1b. Also see Extended Data Fig. 9d. **f**, Schematic flow diagram for the generation of HIS mice. **g**, Representative flow cytometry data from HIS mice showing apparent non-toxicity of aITGB2 CAR T cells against myeloid cells (CD14^+^). All events were used for gating and analysis. Data are representative of *n* = 4 mice and 6 d after CAR T cell treatment. **h**, Quantification of hCD45^+^ data in **g**; *n* = 4 mice. The gating strategy is similar to that shown in Supplementary Information 2e. *P* values were calculated by two-tailed *t*-test (*P* = 0.0008 (aITGB2 CAR versus CD33 CAR) and *P* = 0.0007 (empty CAR versus CD33 CAR)); ***P ≤ 0.001; NS, not significant. **i**, Complete blood count (CBC) profiling of HIS mice treated with aITGB2 CAR T cells on day 5 (data are from *n* = 4 mice). Only aITGB2 CAR T cell manufacturing involved knocking out *ITGB2*. For all the in vitro cytotoxicity assays, the E:T ratio was 1:1 with overnight incubation. All mice used were females; RBC, red blood cells.

**Extended Data Fig. 9:**
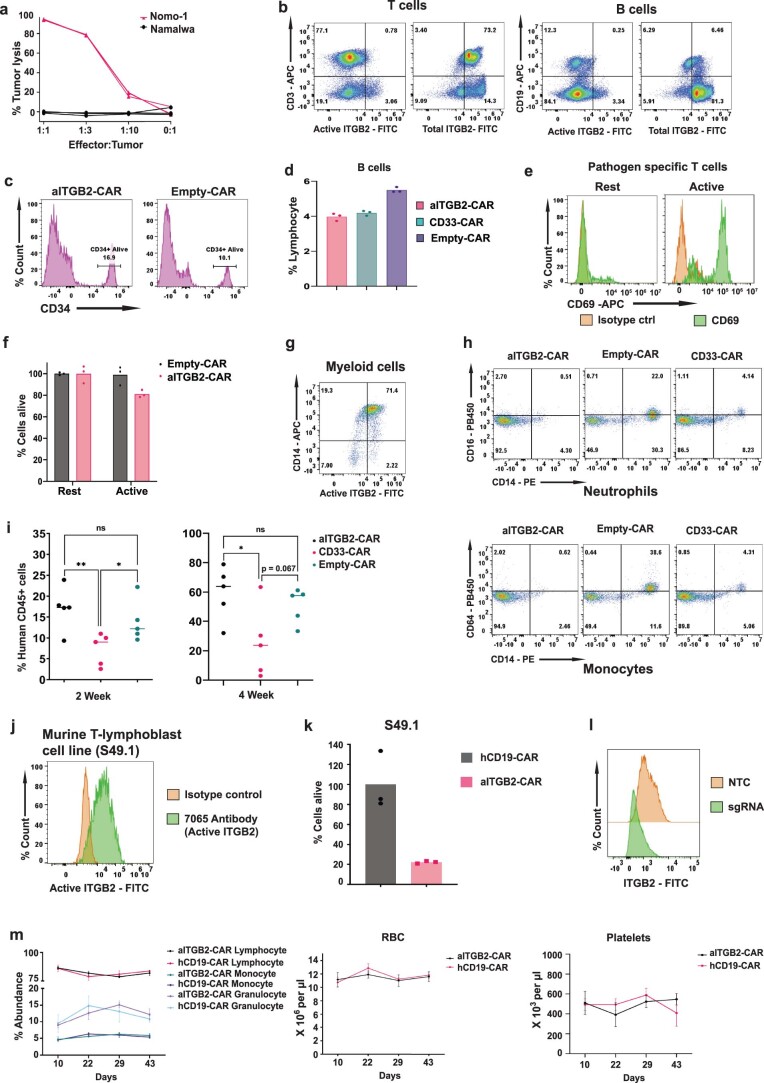
Determining specificity of aITGB2 CAR-T. **a**, Luciferase assay-based cytotoxicity analysis showing no activity of aITGB2 CAR-T vs. Namalwa (B-ALL) line which does not harbor active ITGB2 although it does have total form of ITGB2 (see Fig. 2b). Nomo-1 as the positive control (Data from single experiment performed in triplicate). The luciferase signals of the cytotoxicity assays were normalized against untransduced T-cells of their respective E:T ratios. **b**, Flow cytometry analysis showing absence of active ITGB2 and presence of total ITGB2 in T and B cells (*n* = 3 independent experiments). Cells were gated on single cells for analysis. Gating strategy similar to that shown in Supplementary information 1b. **c**, Flow cytometry analysis showing no discernible impact of aITGB2 CAR-T against CD34+ HSPCs from GM-CSF mobilized peripheral blood. The *y*-axis represents percent count normalized to mode. Gating strategy similar to that shown in Supplementary information 2b (*n* = 1 donor). **d**, Flow cytometry analysis showing non-specific depletion of B cells with aITGB2 and anti-CD33 CAR-T (Representative of *n* = 2 independent experiments with similar results, each experiment performed in triplicate). **e**, Flow cytometry plots showing activation of pathogen-specific T cells using CD69 as a marker. **f**, Quantitative analysis showing no depletion of pathogen-specific T cells with aITGB2 CAR-T when at resting state, but moderate depletion when activated exogenously. Gating strategy shown in Supplementary information 2f (Data from single experiment performed in triplicate). **g**, Flow cytometry analysis showing presence of active ITGB2 in myeloid cells (*n* = 3 independent experiments). Cells were gated on single cells for analysis. Gating strategy similar to that shown in Supplementary information 2c. **h**, Flow cytometry analysis showing cytotoxicity of aITGB2-CAR against neutrophils and monocytes in vitro. Cells were gated on single cells for analysis. **i**, Quantitative plot showing absence of cytotoxicity of aITGB2 CAR-T against human CD45+ cells in HIS mice at noted time points. Peripheral blood samples were used for this analysis. CD33 CAR-T serves as a positive control where significant cytotoxicity was observed (*n* = 5 mice in each arm). Two-tailed t-test were performed for statistical analysis. *[p-value* = *0.009 (2-week, ITGB2-CAR vs CD33-CAR); p-value* = *0.047 (2-week, Empty-CAR vs CD33-CAR); p-value* = 0.035 (4-week, ITGB2-CAR vs CD33-CAR)]; *p ≤ 0.05; **p ≤ *0.01* **j**, Flow cytometry analysis showing cross reactivity of 7065 antibody against the murine ITGB2 on S49.1 cell line. The y-axis represents percent count normalized to mode. Cells were gated on single cells for analysis. Gating strategy similar to shown in Supplementary information 1a. **k**, Quantitative bar plots showing validation of mice aITGB2 CAR-T for its toxicity against murine S49.1 cells. E:T ratio was 2:1 with overnight coculture. hCD19 CAR-T, being specific for human CD19 only, was used as a negative control in murine CAR-T format. Gating strategy shown in Supplementary information 2f. (Data from single experiment performed in triplicate). **l**, Flow cytometry analysis showing knockout efficiency of the mice sgRNA used for knocking out ITGB2 in primary mice T cells (*n* = 1). Cells were gated on single cells for analysis. Flow cytometry gating strategy similar to that shown in Supplementary Information 1a. **m**, Complete blood count profiling of HIS mice treated with aITGB2 CAR-T periodically over 6 weeks (data from *n* = 6 mice). Only aITGB2 CAR-T manufacturing involved knocking out integrin β_2_. All the statistical data in this figure are represented as mean ± SEM. All the mice used in this figure were females.

We next evaluated specificity of our CAR T cells for the active conformation of integrin β_2_. First, we used our Cas9 RNP strategy to confirm that *ITGB2* knockout in Nomo1 cells fully abrogated aITGB2 CAR T cell activity (Fig. 4d,e). To further evaluate conformation specificity, we demonstrated no aITGB2 CAR T cell cytotoxicity versus the B cell leukemia cell line Namalwa, which stains for total integrin β_2_ but not M24 (Fig. 2b and Extended Data Fig. 9a). As a second test, in an overnight assay, we incubated green fluorescent protein (GFP)-labeled aITGB2 CAR T cells with normal donor peripheral blood mononuclear cells (PBMCs). At baseline, we found that aITGB2 showed no cytotoxicity in resting CD3^+^ T cells, which are positive for total integrin β_2_ only (Fig. 5a,e and Extended Data Fig. 9b). However, with PBMC stimulation using ionomycin, lipopolysaccharide and interleukin-2 (IL-2), we found partial depletion of the GFP^–^ (that is non-CAR T cell, derived from PBMCs) T cell population (Fig. 5a–c). Indeed, this partial depletion was consistent with the fraction of T cells expressing active integrin β_2_ after stimulation (Fig. 5b). These results suggest that aITGB2 CAR T cells specifically eliminate target cells displaying the activated conformation of this protein.

### aITGB2 CAR T cells appear to have minimal toxicity in HSPCs

As *ITGB2* only appears to be expressed in hematopoietic cells (Fig. 2f), we focused further toxicity analysis on these populations. By M24 flow cytometry on peripheral blood, we showed that resting T and B cells did not express active integrin β_2_ (Extended Data Fig. 9b). Granulocytes and monocytes appeared strongly positive for active integrin β_2_; however, it is well known that this finding is an artifact of ex vivo activation of these cells after blood collection^45^. We reasoned that evaluating potential aITGB2 CAR T cell cytotoxicity in mature myeloid cells would thus require in vivo studies.

In parallel, we also performed clonogenic assays using GM-CSF-mobilized peripheral blood. Consistent with the lack of active integrin β_2_ on CD34^+^ HSPCs by flow cytometry (Fig. 2c), we found no toxicity against HSPCs after coculture with aITGB2 CAR T cells (Fig. 5d and Extended Data Fig. 9c). Similarly, in PBMCs, we observed no depletion of T cells (Fig. 5e), consistent with our findings in Fig. 5a. Surprisingly, we saw a modest depletion of CD19^+^ B cells compared to that observed with empty control CAR T cells; however, a similar depletion was also seen with anti-CD33 CAR T cells, arguing against target specificity (Extended Data Fig. 9d). We also tested our CAR T cells versus pathogen-specific T cells, where we found modest depletion only after exogenous activation (Extended Data Fig. 9e,f), consistent with donor T cells (Fig. 5c). As expected, based on known artifactual integrin β_2_ activation (Extended Data Fig. 9g), and confirming in vitro potency of aITGB2 CAR T cells versus primary cells, we found strong depletion of monocytes and neutrophils (Extended Data Fig. 9h).

We next moved into a HIS mouse model, where CD34^+^ HSPCs isolated from GM-CSF-mobilized peripheral blood were intravenously implanted into busulfan-treated NOD *scid* gamma (NSG)-SGM3 mice^46^ (Fig. 5f). Mice were monitored by peripheral blood draw at 8 weeks after implantation to confirm hematopoietic engraftment, as assessed by ≥1.5% circulating human CD45^+^ cells. At this time, we treated all engrafted mice (16 of 25 total implanted) with aITGB2, anti-CD33 or empty CAR T cells, and 6 d later, we killed the mice and analyzed peripheral blood. Although rigorous quantification of CD14^+^ cells was not possible due to high variability in myeloid engraftment at the time of CAR T cell treatment, we found no discernible depletion after treatment with aITGB2 CAR T cells (Fig. 5g). Importantly, we found a significant depletion of total human CD45^+^ cells after treatment with CD33 CAR T cells (Fig. 5h). Long-term monitoring up to 4 weeks found similar results (Extended Data Fig. 9i). These results recapitulated the expected toxicity of targeting CD33 given its expression on HSPCs and myeloid cells. By contrast, human CD45^+^ cells continued to expand in mice treated with either aITGB2 or empty CAR T cells (Fig. 5h).

Furthermore, we probed the 7065 antibody clone and found that it was cross-reactive with mouse activated integrin β_2_ (Extended Data Fig. 9j). To further assess toxicity, we thus performed a complete blood count analysis of mouse blood from our HIS mouse study. Five days after aITGB2 CAR T cell treatment, we found no depletion of any mouse PBMC types (Fig. 5i). We also performed an analogous study with a non-tumor-bearing immunocompetent mouse model (C57BL/6), challenged them with mouse aITGB2 CAR T cells and found a similar safety profile (Extended Data Fig. 9k–m). Taken together, these results suggest that treatment with aITGB2 CAR T cells may carry minimal toxicities to bystander immune cells, thus underscoring a promising safety profile.

### aITGB2 CAR T cells are efficacious against PDX models of AML

**Fig. 6:**
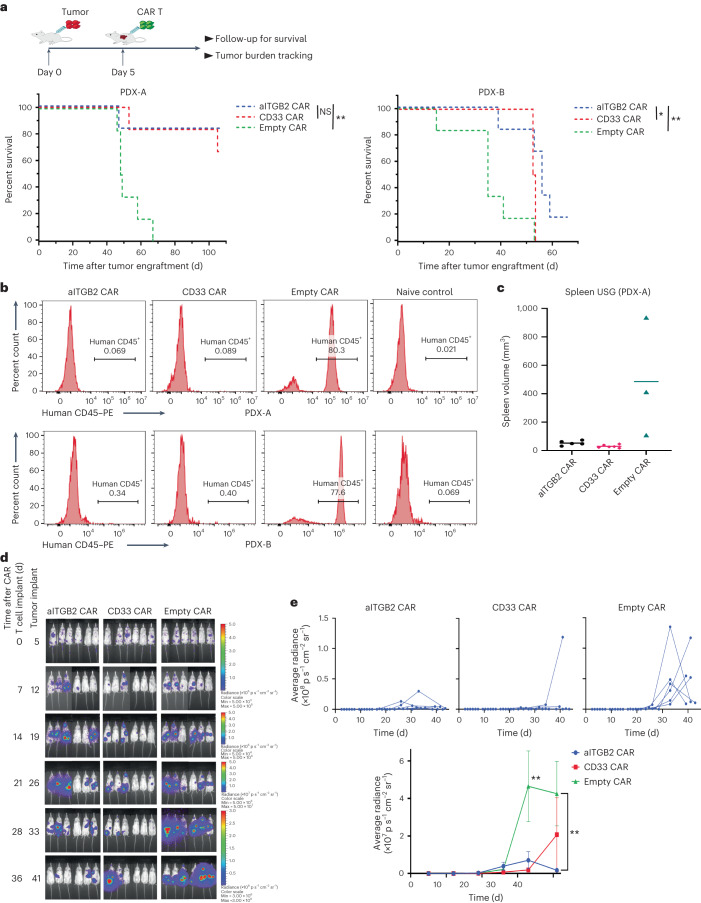
Efficacy of aITGB2 CAR T cells against AML models in vivo. **a**, Survival of NSG mice implanted with two independent AML PDX models and treated with aITGB2, anti-CD33 or empty CAR T cells; *n* = 6 mice per arm. In total, 2 × 10^6^ AML tumor cells were injected on day 0, and 5 × 10^6^ CAR T cells were injected on day 5. *P* values were determined by log-rank test (*P* = 0.009 (ITGB2 CAR T cells versus empty CAR T cells, PDX-A); *P* = 0.0068 (ITGB2 CAR T cells versus empty CAR T cells, PDX-B); *P* = 0.044 (CD33 CAR T cells versus ITGB2 CAR T cells, PDX-B)); **P ≤ 0.01; *P ≤ 0.05. **b**, Flow cytometry histogram plots of peripheral blood draws showing tumor burden at 8 weeks after tumor injection for PDX-A and at 3.5 weeks for PDX-B (also see Extended Data Fig. 10a,b). Naive control mice have no human cells (AML tumor or CAR T cells) injected and were used to assess background noise in the flow cytometry assay. The *y* axis represents percent count normalized to mode. The gating strategy is similar to that shown in Supplementary Information 2e. Data are representative of *n* = 6 mice per arm. Plots of only live animals are provided at the respective time points. **c**, Spleen ultrasonography (USG) from animals treated with empty CAR T cells compared to animals treated with CD33 or aITGB2 CAR T cells. All mice alive at day 49 after tumor implantation were scanned. Data are from mice still surviving at this time; *n* = 5 (aITGB2), *n* = 6 (CD33) and *n* = 3 (empty). **d**, BLI imaging showing the efficacy of aITGB2 CAR T cells against the intravenously implanted AML cell line Nomo1 (*n* = 6 mice per arm). **e**, Quantitative analysis of bioluminescence intensity of the mice in **d** plotted individually (*n* = 6 mice). A two-tailed Mann–Whitney test was used for statistical analysis of bioluminescence quantification (*P* = 0.0087 (day 33) and *P* = 0.0022 (day 41) ITGB2 CAR T cells versus empty CAR T cells); **P ≤ 0.01. Only aITGB2 CAR T cell manufacturing involved knocking out *ITGB2*. All statistical data are represented as mean ± s.e.m. All mice used in **a**–**c** were females, and those in **d–e** were male.

**Extended Data Fig. 10:**
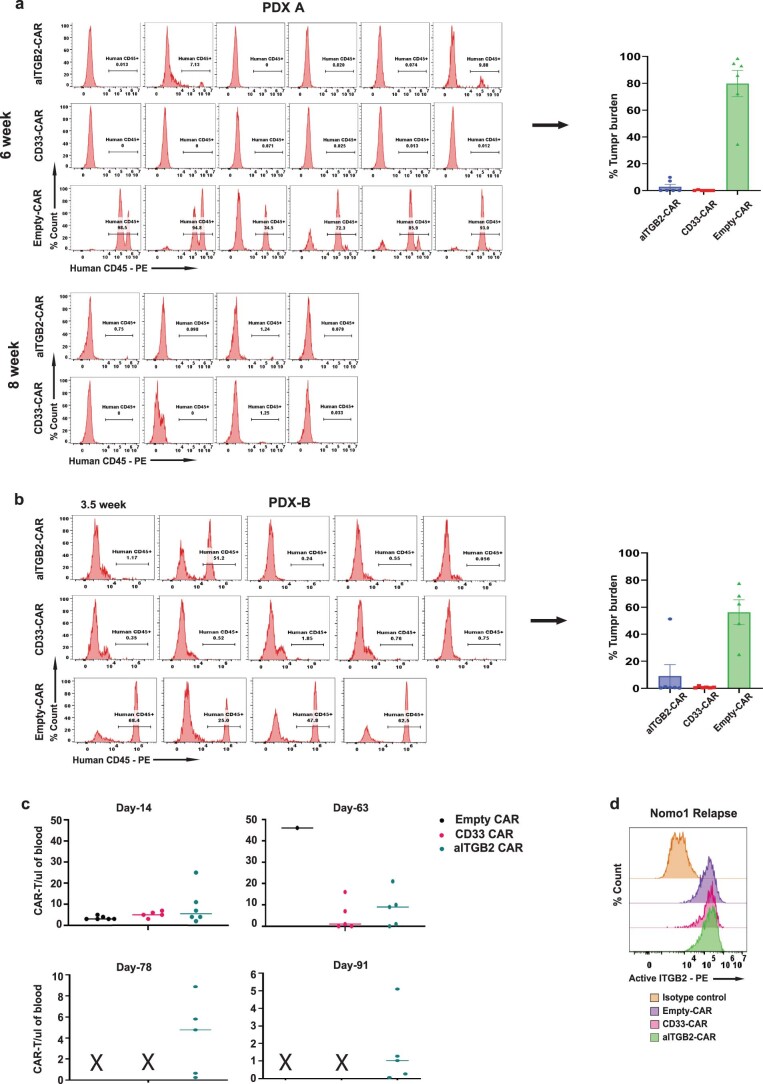
aITGB2 CAR-T efficacy in PDX models. **a**, Flow cytometry analysis and bar graph of peripheral blood draw showing tumor burden at 6 and 8 weeks post tumor injection of PDX-A. The *y*-axis represents percent count normalized to mode. Cells were gated on single cells for analysis. Representative of data from *n* = 6 mice per arm; plots of only mice alive at designated time point. **b**, Flow cytometry analysis and bar graph of peripheral blood draw showing tumor burden at 3.5 weeks post tumor injection of PDX-B. The *y*-axis represents percent count normalized to mode. Cells were gated on single cells for analysis. Representative of data from *n* = 6 mice per arm; plots of only mice alive at designated time point. **c**, Plots showing expansion and persistence of aITGB2 CAR-T in mice peripheral blood, analyzed periodically for 3 months (*n* = 6 mice per arm, subsequent data points were from mice alive at that time point). **d**, Flow cytometry analysis showing active ITGB2 density of tumor cells harvested from relapse Nomo-1 mice model (*n* = 4 mice per condition). The *y*-axis represents percent count normalized to mode. Cells were gated on human CD45+ cells for analysis. Gating strategy similar to that shown in Supplementary information 2e. Only aITGB2 CAR-T manufacturing involved knocking out integrin β_2_. All the statistical data in this figure are represented as mean ± SEM. All the mice used in this figure were females.

Finally, we evaluated in vivo efficacy of aITGB2 CAR T cells. We established two separate monocytic leukemia PDXs obtained from PRoXe^35^ via intravenous implantation in NSG mice. Both samples appeared to express active integrin β_2_ as determined by flow cytometry with M24 (Extended Data Fig. 3c). Five days after implantation of 2 × 10^6^ PDX AML cells, we administered 5 × 10^6^ empty, aITGB2 or CD33 CAR T cells. Tumor burden was monitored by peripheral blood draw and evaluation of human CD45^+^ cells and/or ultrasonography for spleen size (Fig. 6b,c and Extended Data Fig. 10a,b). In both models, we saw marked elimination of human CD45^+^ cells as well as decreased spleen size in mice treated with aITGB2 or CD33 CAR T cells, with prominent outgrowth of tumor cells in empty control (Fig. 6b,c). In both models, survival was significantly improved in mice treated with aITGB2 CAR T cells compared to those treated with empty control CAR T cells, and survival was similar between mice treated with aITGB2 CAR T cells and those treated with anti-CD33 CAR T cells (Fig. 6a). aITGB2 CAR T cells also showed favorable expansion and persistence properties in the peripheral blood (Extended Data Fig. 10c). In a Nomo1 cell line xenograft mouse model implanted in NSG mice, we again noted improved tumor control over empty CAR T cells as well as similar efficacy of aITGB2 CAR T cells and anti-CD33 CAR T cells (Fig. 6d,e). However, in this aggressive model, neither tested CAR T cells could lead to complete tumor eradication. Toward initial investigation of a possible mechanism of relapse after aITGB2 CAR T cell treatment, we performed flow cytometry on mouse spleens after killing mice at day 42 after tumor implantation. Gating on human CD45^+^ AML blasts, we found no evidence of downregulation or loss of activated integrin β_2_ (Extended Data Fig. 10d). This initial experiment suggests that loss of the activated conformation of integrin β_2_ may not be an immediate mechanism of resistance to our structurally selective targeting.

## Discussion

Our structural surfaceomics approach presented here, integrating XL–MS with cell surface glycoprotein enrichment, is a technology designed to expand the targetable space of cell surface immunotherapy antigens. This strategy may also carry promise in applications in other basic or translational science fields.

However, we acknowledge that our current methodology carries limitations. The first limitation is sample input. XL–MS has traditionally required large sample inputs (10^9^ cell scale) and extensive mass spectrometer time for analysis. These limitations led us to focus our initial efforts on a single AML cell line with multiple XL–MS approaches. However, future optimization of enrichable cross-linkers, alternative cross-linker reactivities and/or further technological MS advances may enable broader-scale profiling of both tumor and normal cells or even primary samples. The second limitation is the analysis and validation of potential targets. In the current study, we manually compared identified cross-links to PDB structures to find targets of interest. Future work will aim to develop automated computational structural analysis to identify the most promising targets for workup. Additional investigation will be required to evaluate constraints of our method based on surface protein glycosylation or other biophysical parameters. Further work will also clarify the underlying reasons for limited overlap between cross-links found by DSSO and PhoX, which may relate to acquisition on different mass spectrometers, different analysis pipelines and/or different radius of reactivity. In terms of validation, we chose to first investigate integrin β_2_ in depth because we had flow cytometry and biochemical (that is, Mn^2+^) tools to probe its conformation status. For other potential targets, these tools will not exist a priori. We thus anticipate future efforts to develop alternative strategies (for example, ‘disulfide locking’, as used in structural biology studies of membrane proteins^47^) to generate putative tumor-selective conformations for recombinant antibody selection and subsequent validation.

The active conformation of integrin β_2_ carries promise compared to other AML immunotherapy targets given a potentially improved safety profile. Although we anticipate some unwanted activity toward activated myeloid or T cells, we predict that this toxicity will be markedly lower than CD33, CD123 or CLL-1 that are expressed widely on mature myeloid cells^48^. CD70 is also expressed on activated T cells, but this property has not hindered its clinical development as an AML CAR T cell target (NCT04662294). Our ex vivo results also suggest that depletion of activated T cells may be limited. Future studies in immunocompetent mouse models of inflammation or infection may be useful to evaluate the impact of aITGB2 CAR T cell depletion of activated hematopoietic cells.

In terms of efficacy, like many other AML targets^12,49^, we observed heterogeneity of active integrin β_2_ on primary human tumor samples. Stratification of individuals by flow cytometry for this target may thus be required for any future clinical studies. Furthermore, PDX-B, despite higher aITGB2 expression, relapsed more rapidly than PDX-A after either aITGB2 or CD33 CAR T cell treatment. Future PDX modeling will further delineate the relationship between antigen density and in vivo aITGB2 CAR T cell efficacy. The favorable safety profile of aITGB2 CAR T cells may also create future opportunities for multitargeting CARs targeting two or more antigens with complementary but heterogeneous tumor expression patterns. Future antibody engineering efforts, or incorporation of emerging engineered T cell antigen receptor designs^44^, may be able to enhance the efficacy of aITGB2 CAR T cells targeting tumors with low antigen levels.

In conclusion, our studies demonstrate a potential systematic approach to identify and target conformation-specific antigens in cancer. Humanized aITGB2 CAR T cells, discovered via this approach, stand as a promising proof-of-principle therapeutic warranting further preclinical evaluation in AML and a pathway for other applications of structurally directed immunotherapeutic targets.

## Methods

### Ethics statement

All primary AML samples used were obtained under Institutional Review Board-approved protocols by the University of California San Francisco (UCSF), Committee on Human Research and following the Declaration of Helsinki. Informed consent was obtained from participants for research purposes, although for this study, all samples were fully deidentified and could not be linked back to relevant clinical information, including participant sex. All mouse experiments were conducted in accordance with an approved protocol by the UCSF Institutional Animal Care and Usage Committee. Mice were housed in the UCSF Animal Care Facility Laboratory Animal Resource Center at the Helen Diller Family Cancer Center at UCSF Mission Bay. Animals were housed in an individual specific pathogen-free suite with up to five mice per ventilated cage and ad libitum access to food and water. The suite was maintained on a 12-h light/12-h dark cycle with controlled temperature (~19–23 °C) and humidity (30–70%) conditions.

### Cell lines, PDX and human samples

Nomo1 and BV-173 cell lines were obtained from DSMZ, and THP-1, HL-60, MV-4-11, Jurkat, U937, Namalwa and S49.1 cell lines were obtained from ATCC. All cell lines were grown in RPMI-1640 medium (Gibco, 11875093) supplemented with 20% fetal bovine serum (FBS; BenchMark, Gemini, 100-106) and 100 U ml^–1^ penicillin–streptomycin (UCSF Cell Culture Facility). All cells were grown at 37 °C with 5% CO_2_. All AML PDXs were procured from the PRoXe at Dana–Farber Cancer Institute under an appropriate materials transfer agreement. Primary AML samples were obtained from the UCSF Hematologic Malignancies Tissue Bank and the Pediatric Hematopoietic Tissue Cell Bank.

### Cross-linking and cell surface labeling

DSSO-based (Sigma-Aldrich, 909602) XL–MS involving HpHt and PhoX-based (Thermo Fisher Scientific, A52286) XL–MS were each performed with 2.4 × 10^9^ cells (in batches of 6 × 10^8^). Cells were collected and washed (300*g*, 5 min) three times with PBS. Cross-linker DSSO or PhoX predissolved in DMSO (Sigma-Aldrich, 276855) was then added to the cells at a final concentration of 10 mM and incubated at room temperature for 45 min. Cross-linking was followed by biotinylation of cell surface proteins. Briefly, N-linked sugar residues of the cells were oxidized with 1.6 mM sodium metaperiodate (VWR, 13798-22) for 20 min at 4 °C, followed by installation of biotin on those residues using 10 mM aniline (Sigma-Aldrich, 242284) and 1 mM biocytin hydrazide (Biotium, 90060) for 90 min at 4 °C. The cells were then washed with PBS, snap-frozen and stored at −80 °C until further processing.

### Cell surface proteomics sample preparation

Frozen cell pellets were thawed on ice and resuspended in 1 ml of RIPA lysis buffer (MilliporeSigma, 20-188) with Halt protease inhibitor (Thermo Scientific, 78430) and 1 mM EDTA (Invitrogen, 15575-038), followed by sonication for lysis. Lysates were centrifuged at 17,000*g* for 10 min at 4 °C, and clarified supernatant was mixed with 0.5 ml of Neutravidin beads (Thermo Scientific, PI29204), followed by incubation at 4 °C for 2 h in an end-to-end rotor. Beads were washed extensively by vacuum manifold (Promega) with 50 ml of RIPA lysis buffer + 1 mM EDTA, 50 ml of PBS + 1 M NaCl and 50 ml of 2 M urea (VWR, 97063-798) + 50 mM ammonium bicarbonate. Beads were resuspended in 50 mM Tris (pH 8.5) + 4 M urea + 10 mM TCEP (Gold Biotechnology, TCEP10) and 20 mM iodoacetamide (VWR, 97064-926). Ten micrograms of trypsin-LysC (Thermo Scientific, PRV5073) mix was added for on-bead digestion with simultaneous reduction and alkylation. After 2 h, the mixture was diluted to 1.5 M urea using 50 mM Tris (pH 8.5) and incubated overnight (16–20 h). Beads were eliminated by centrifugation, and the resulting supernatant was acidified with 0.5% trifluoroacetic acid (TFA). Peptides were desalted using a SOLA HRP Column (Thermo Scientific, 60109-001) and eluted with 50% acetonitrile (ACN) + 0.1% formic acid (FA).

### IMAC purification for PhoX

Dry peptides were reconstituted in 80% ACN + 0.1% TFA. Superflow Ni-NTA beads were stripped off using EDTA and reloaded with FeCl_3_ (Sigma-Aldrich, 451649) on a polyprep chromatography column (Bio-Rad, 7326008). Fe^3+^-loaded beads were transferred to C18 tips (Nest Group, SEM SS18V.25) and incubated for 4–6 min, followed by washing with 0.5% FA. The bound peptides were eluted from beads with 0.5 M potassium phosphate buffer (pH 7.4). Peptides eluted from beads were bound to C18 chromatographic material of the Nest tips, washed three times with 0.5% FA and eluted with 50% ACN + 0.1% FA.

### SEC

Size-based fractionation of peptides was performed using a Superdex Peptide 3.2/300 (GE Healthcare) column and high-performance liquid chromatography (HPLC; Agilent 1260 Infinity II). Dried peptides were reconstituted in the mobile phase of 30% ACN + 0.1% TFA and loaded on the column. Run time was 90 min at a flow rate of 50 µl min^–1^, and 45 fractions (2 min per fraction) were collected. Fractions associated with the desired molecular weight were dried down in a SpeedVac and stored at −80 °C for MS analysis.

### LC–MS and data-dependent acquisition analysis

Peptide samples prepared for building the Nomo1 cell surfaceome custom database were loaded on to the EASY-Spray nanocolumn (Thermo Scientific, ES900) installed on a Dionex Ultimate 3000 NanoRSLC instrument coupled with a Q-Exactive Plus mass spectrometer (Thermo Scientific). Peptides were separated primarily over a 313-min gradient ranging from 2.4% to 32% ACN with a flow rate of 0.3 μl min^–1^. MS scans were performed from *m*/*z* 299 to 1,799 at a resolution of 70,000 full-width at half-maximum (FWHM) at *m*/*z* 200 with an automatic gain control (AGC) target of 3 × 10^6^ and maximum injection time of 100 ms. The resolution for MS/MS scans was set to 17,500 FWHM at an *m*/*z* of 200 with an AGC target of 2 × 10^5^ and maximum collision-induced dissociation of 200 ms. Normalized collision energies of 27%, 30% and 33% in stepped higher collision-induced dissociation mode were used for fragmentation of the top 15 most intense precursor ions with an isolation window of 1.7 *m*/*z*. Peptides with a charge state of 2^+^ or higher were considered for MS/MS. Dynamic exclusion was set to 20 s.

MS-generated .raw files were processed using MSFragger^50^ within FragPipe v14.0 with default settings unless stated otherwise. Briefly, the spectral data were searched against the human proteome database (UniProt, downloaded 11 May 2021; 20,395 entries). The contaminant and decoy protein sequences were added to the search database using the inbuilt feature of the FragPipe v14.0 pipeline downstream statistical analysis. The inbuilt tools PeptideProphet and ProteinProphet were used for statistical validation for 1% false discovery rate (FDR).

### HpHt-based fractionation of DSSO cross-linked peptides

The SEC fractions 13 and 14, which are enriched with DSSO cross-linked peptides (Extended Data Fig. 1b), were further fractionated by HpHt as described previously^23^. Briefly, the HpH tip was constructed in a 200-µl pipette tip by packing C8 membrane (Empore 3M) and 5 mg of C18 solid phase (3 μm; Durashell, Phenomenex). The column was sequentially washed with three different solvents/solutions: methanol, ACN and ammonia water (pH 10; 90 µl each). Samples were loaded on the column, followed by washing with 90 µl of ammonia water (pH 10) and a series of ammonia water containing increasing concentration of ACN (6, 9, 12, 15, 18, 21, 25, 30, 35 and 50%). The fractions with 25, 30, 35 and 50% ACN were combined with fractions containing 6, 9, 12 and 21% ACN, respectively, for MS analysis.

### LC–MS^3^ analysis of DSSO cross-linked peptides

The SEC–HpHt fractions were subjected to LC–MS^3^ analysis using an UltiMate 3000 RSLC nano-HPLC system coupled to an Orbitrap Fusion Lumos mass spectrometer (Thermo Scientific), as described previously^23^. Peptides were separated by reversed-phase LC (50 cm × 75 μm Acclaim PepMap C18 column, Thermo Scientific) with over an 87-min gradient of ACN (4% to 25%) at a flow rate of 300 nl min^–1^. Initial survey (MS^1^) scans were measured in the Orbitrap with a scan range from 375 to 1,800 *m*/*z*, a resolution of 60,000 FWHM and an AGC target of 4 × 10^5^ with a maximum injection time of 75 ms at top speed per 4 s of cycle time. Ions with a charge of 4^+^ or greater were selected for MS^2^ and subjected to fragmentation using collision-induced dissociation with a normalized collision energy of 23%. For MS^2^ scans, the scan range was set to auto mode, with a resolution of 30,000 FWHM, an AGC target of 5 × 10^4^, a precursor isolation width of 1.6 *m*/*z* and a maximum injection time of 100 ms. A targeted inclusion on ions with a mass difference corresponding to the difference in alkene and thiol DSSO fragments (31.9721 Da) was used to select precursors for MS^3^ analysis. For MS^3^ scans, higher collision-induced dissociation was used with a normalized collision energy of 28%, the AGC target was set to 2 × 10^4^, and the maximum injection time was set to 125 ms.

### Identification of DSSO cross-linked peptides

Peak lists were extracted from the LC–MS^*n*^ raw files using the in-house software PAVA (UCSF), and the extracted MS^3^ spectra were searched against a SwissProt database (2021.10.02 version; 20,387 entries) concatenated with its randomized decoy sequences using Protein Prospector (v.6.3.5). The mass tolerances allowed were ±20 ppm for precursor ions and 0.6 Da for fragment ions. The database search was performed with trypsin as a protease with a maximum of three allowed missed cleavages. Cysteine carbamidomethylation was set as the fixed modification. Variable modifications included N-terminal protein acetylation, methionine oxidation and N-terminal conversion of glutamine to pyroglutamic acid. Additionally, three specific modifications resulting from DSSO were included in the search: thiol (C_3_H_2_SO, +86 Da), alkene (C_3_H_2_O, +54 Da) and sulfenic acid (C_3_H_4_O_2_S, +104 Da)^19^. The in-house software XL-Tools was used to automatically identify, summarize and validate cross-linked peptides based on Protein Prospector database search results and MS^*n*^ data. No decoy hits were found after the integration of MS^1^, MS^2^ and MS^3^ data.

### Development of the MS^3^-based XL–MS analysis tool

We developed Ving to assess the MS^2^/MS^3^-based cleavable cross-linking database search results to produce a set of cross-linked spectrum matches (CSMs; Extended Data Fig. 1a). Ving is open-source, publicly available software and conceptually based on our previously published search methodology^19^ but now adapted to the proteomics analysis suite, the TPP. Ving input consists of spectral data in mzML format^51^ and database search results in PepXML format^52^.

Ving functions by parsing data to create spectral groups (SGs) consisting of MS^2^ and MS^3^ data originating from a single precursor ion. Next, database search results from the MS^2^ and MS^3^ scan events, performed using the TPP^22^ as described previously, are added to each SG. A series of thresholds categorize each SG to determine probable CSMs after peptide sequence assignments to all MS^2^ and MS^3^ spectra within all groups. First, MS^2^ peptide sequence assignments with a probability of >0.8 are labeled as single, non-linked peptide spectrum matches (PSMs). Evidence of an internal lysine residue with a hydrolyzed cross-linker mass refines the classification to dead end or monolinked PSMs. For the remaining SGs, the MS^3^ peptide assignments and probabilities are evaluated. If the SG has multiple MS^3^-level peptide sequence identifications with a probability of >0.8 and a cross-linker-modified lysine residue, those sequences are further evaluated as candidate CSMs. If two peptide sequence masses plus the cross-linker mass sum together to match the mass of the original precursor ion, then the group is classified as a CSM. If none of the peptide sequences sum to the precursor mass, then the SG is classified as an incomplete CSM. If the SG has one or zero MS^3^-level peptide sequences with a probability of >0.8, the group is classified simply as unknown PSMs. After all SGs are evaluated, a human-readable text summary is reported to the user.

### LC–MS analysis of PhoX cross-linked peptides

PhoX cross-linked peptide samples were analyzed on a timsTOF Pro mass spectrometer (Bruker Daltronics) as described previously^53^. Briefly, peptides from each SEC fraction 9–24 (Extended Data Fig. 1c) were loaded on to the column operated using an UltiMate 3000 RSLC nano-HPLC system (Thermo Scientific), and eluted peptides were analyzed with the timsTOF Pro mass spectrometer using a CaptiveSpray source (Bruker Daltonics). Peptides were first trapped on a C18 precolumn (Acclaim PepMap 100, 300 μm × 5 mm, 5 μm, 100 Å; Thermo Scientific), and eluted peptides were subsequently separated on a μPAC 50 column (PharmaFluidics) over 180 min with an ACN gradient ramping up from 3% to 35%, during which the flow rate changed from 900 to 600 nl min^–1^ for the first 15 min, followed by a constant flow rate of 600 nl min^–1^.

For MS analysis with the timsTOF Pro mass spectrometer, the mobility-dependent collision energy ramping settings were 95 eV at an inversed reduced mobility (1/*k*_0_) of 1.6 V s^–1^ cm^–2^ and 23 eV at 0.73 V s^–1^ cm^–2^. Collision energies were interpolated linearly between the two 1/*k*_0_ values and were kept constant above or below. TIMS scans were not merged, and the target intensity per individual parallel accumulation serial fragmentation precursor ion was kept at 20,000 (with an intensity threshold of 1,000). The mobility range of each scan was kept between 0.6 and 1.6 V s^–1^ cm^–2^ with a ramp and accumulation time of 166 ms, and the mass range for MS and MS/MS was set to 100–1,700 *m*/*z*. The number of parallel accumulation serial fragmentation MS/MS scans triggered was 14 per cycle (2.57 s), with a maximum of seven allowed precursors per mobilogram. The precursor ion charge states selected for fragmentation ranged between 3^+^ and 8^+^, and the isolation width was 2 Th for precursor *m*/*z* up to 700 and 3 Th for precursor *m*/*z* > 800, in between which it was ramped linearly. The active exclusion was set to 0.4 min (mass width of 0.015 Th and 1/*k*_0_ width of 0.015 V s^–1^ cm^–2^).

### TimsTOF MS data analysis

TimsTOF MS data were converted to .mgf format using MSConvert^54^. The .mgf files were then processed for identification of cross-linked peptides using pLink-2 (ref. ^55^) with default settings unless stated otherwise. All files were searched against the Nomo1 cell surfaceome-specific custom database (5,280 entries) generated from regular data-dependent acquisition analysis based on prior CSC data (that is, non-cross-linked). For pLink-based cross-linked peptide analysis, trypsin was set as the protease, allowing three missed cleavages. Cysteine carbamidomethylation was set as the fixed modification with methionine oxidation and N-terminal acetylation as the variable modifications. The search was performed with a ±20 ppm mass tolerance window for precursor and fragment ions, and results were reported at 1% FDR.

### Flow cytometry

Immunostaining of cells was performed as per the instructions from the antibody vendor unless stated otherwise. Briefly, 1 × 10^6^ cells were resuspended in 100 µl of FACS buffer (PBS + 2% FBS) with 1 µg of antibody added. Cells were incubated at 4 °C for 10–15 min and washed three times with FACS buffer. For staining the active form of integrin β_2_, the antibody incubation step was performed at 37 °C for 1 h. For staining primary AML cells for activated integrin β_2_, the FACS buffer was RPMI-1640 + 5% FBS + 2% bovine serum albumin (BSA) + 50 µg ml^–1^ DNase I (Gold Biotechnology, D-301-500). For other primary antibody cell stainings, FACS buffer was D-PBS + 5% FBS + 2% BSA + 5 mM EDTA + 50 µg ml^–1^ DNase I with Human Trustain (Biolegend, 422302). Compensation used UltraComp eBeads Compensation Beads (Invitrogen, 01-2222-42). All antibodies were diluted 1:20 unless stated otherwise. Flow cytometry analysis was performed on the CytoFLEX platform (Beckman Coulter), and data were analyzed using FlowJo_v10.8.1.

### Phage display selections

A synthetic, phage-displayed Fab library^42^ was selected for binding to either integrin β_2_/integrin α_M_ (R and D 4047-AM, antibodies 7062 and 7065) or integrin β_2_/integrin α_L_ (R and D 3868-AV, antibodies 7060 and 7341) recombinant protein complexes. Briefly, integrin β_2_ recombinant protein complexes were immobilized on Maxisorp Immuno plates (Thermo Fisher, 12-565-135) and used for positive binding selections with library phage pools that were first exposed to neutravidin-coated wells to deplete nonspecific binders. After four rounds of binding selections, clonal phage was prepared and evaluated by phage ELISA and sequencing as previously described^42^.

### Antibody production

Antibodies were produced using the human Expi293 expression system (Thermo Fisher). Expi293 cells (in a volume of 2 ml) were transiently transfected with construct DNA using FectoPro transfection reagent (Polyplus Transfection, 101000014). Following a 5-d expression period, antibodies were purified using rProteinA Sepharose (GE Healthcare) and stored in phosphate buffer (50 mM NaH_2_PO_4_, 75 mM Na_2_HPO_4_, 100 mM H_3_PO_4_ and 154 mM NaCl).

### BLI binding assays

Binding of human integrin β_2_ antibodies was tested against three different integrin β_2_ complexes, including integrin β_2_/integrin α_M_ (R and D 4047-AM), integrin β_2_/integrin α_X_ (R and D 5755-AX) and integrin β_2_/integrin α_L_ (R and D 3868-AV). To determine binding kinetic parameters, BLI was performed on an Octet HTX instrument (Sartorius) at 1,000 r.p.m. and 25 °C. All proteins were diluted in assay buffer (PBS, 1% BSA and 0.05% Tween 20). Tested and negative-control antibodies at 2 µg ml^–1^ were first captured on AHQ biosensors to achieve binding signals of 0.8–1.3 nm. Unoccupied Fc-binding sites on the antibody-coated sensors were subsequently quenched with 20 µg ml^–1^ Fc protein. After equilibration with assay buffer, biosensors were dipped for 600 s into wells containing a fivefold serial dilution of integrin β_2_ complexes (association phase), followed by transfer back into assay buffer for an additional 600 s (dissociation phase). Assay buffer alone served as a negative control. Binding response data were reference subtracted and globally fitted with a 1:1 binding model using ForteBio’s Octet Systems software v9.0.

### Nonspecific ELISA panel

The ELISA protocol to assess interactions between the antibodies and unrelated macromolecules was performed as described previously^56^. The tested antigens included cardiolipin (50 μg ml^–1^; Sigma, C0563), keyhole limpet hemocyanin (5 μg ml^–1^; Sigma, H8283), lipopolysaccharide (10 μg ml^–1^; InvivoGen, tlrl-eblps), single-stranded DNA (1 μg ml^–1^; Sigma, D8899), double-stranded DNA (1 μg ml^–1^; Sigma, D4522) and insulin (5 μg ml^–1^; Sigma, I9278). In addition, binding of each antibody was also tested against empty wells (BSA-only control) and wells containing goat anti-human Fc (positive control, 1 µg ml^–1^; Jackson, 109-005-098). Antigens were coated at 30 µl per well in 384-well Maxisorp plates and incubated at 4 °C overnight. Plates were blocked with 0.5% BSA for 1 h at room temperature and washed with PBS + 0.05% Tween 20. Antibodies were added at 100 nM and allowed to bind for 60 min at room temperature. Plates were washed with PBS + 0.05% Tween 20, and binding was detected with anti-κ-horseradish peroxidase (1:5,000; Southern Biotech, 2060-05) and developed with TMB substrate (KPL (Mandel), KP-50-76-03).

### Lentiviral construct generation and production

Second-generation lentivirus constructs were used for transducing CAR expression cassettes in human T cells. Packaging plasmids used were pCMV delta R8.2 and pMD2.G. The transfer vector carrying the CAR expression cassette was pHR lentiviral vector with the SFFV promoter.

All transfer lentiviral plasmid constructs were generated using NEBuilder HiFi DNA Assembly master mix (New England Biolabs, E2621L) as per the vendor’s instructions with minor modifications. The DNA fragments containing the binder (scFv) sequence along with the 40-base pair vector-compatible flanking region for Gibson assembly were procured from Twist Bioscience. Target CAR plasmid backbone was linearized with BamHI-HF (New England Biolabs, R3136T). Stbl3 competent *Escherichia coli* cells (QB3 MacroLab, University of California, Berkeley) were used for transformation, and colonies obtained were screened by Sanger sequencing (Genewiz).

Lenti-X cells cultured in DMEM and plated 1 d before in a six-well plate (1 million cells per well) were transfected with the lentiviral plasmid constructs using polyethylenimine (Polysciences, 40,000 Da molecular weight, 24765-100). The spent media in cultures containing the lentivirus were collected 72 h after transfection. Twelve microliters of the stock concentration of polyethylenimine (2.5 mg ml^–1^) was used for transfecting 3 µg of plasmid per well. In total, 1.35 µg, 0.165 µg and 1.5 µg of lentiviral plasmids pCMV delta R8.2, pMD2.G and pHR (transfer vector), respectively, were used per well.

### Human primary T cell isolation

Primary T cells were isolated from LeukoPaks (Stem Cell Technologies, 200-0092). CD8^+^ and CD4^+^ cells were isolated separately using an EasySep human CD8^+^/CD4^+^ T cell isolation kit (StemCell, 17952 (CD4); StemCell, 17953 (CD8)) based on magnetic bead separation. Isolated cells were stored frozen with 10% DMSO (MP Biomedicals, 196055). In total, primary T cells from five different donors were used for in vitro and in vivo studies.

### Human CAR T cell generation

T cells were thawed and grown in T cell medium consisting of Optmizer CTS medium (Gibco, A10221-01) + CTS supplement (Gibco, A10484-02) + 5% human AB serum (Valley Biomedical, HP1022) + penicillin/streptomycin + GlutaMAX (Gibco, 35050-061). Recombinant human IL-7 (Peprotech, 200-07) and IL-15 (Peprotech, 200-15; a final concentration of 10 ng ml^–1^ each) were freshly added to cells every 2–3 d. For manufacturing CAR T cells, primary T cells (CD4^+^ or CD8^+^) were thawed and cultured overnight. For aITGB2 CAR T cells, the cells were then additionally nucleofected with ribonuclease complex of *ITGB2* sgRNA and Cas9 using a P3 Primary Cell 4D-Nucleofector X kit S (Lonza, V4XP-3032) and 4D-Nucleofector (Lonza) with its built-in program EO-115. Cells were then stimulated with 20 µl of CD3/CD28 Dynabeads (Thermo Fisher Scientific, 11131-D) per 1 million cells on day 0. The following day, lentivirus carrying the CAR expression cassette was added to the cells. The virus was withdrawn from the culture after 24 h, followed by two to three rounds of PBS washes by centrifugation. Dynabeads were magnetically withdrawn on day 4. On day 6 or 7, CAR^+^ cells were sorted by magnetic-activated cell sorting using the Myc tag of the CAR constructs with biotinylated c-Myc antibody (Milteni Biotec, 130-124-877). CAR T cells were used for studies within days 10–14 of culture.

### CAR T cell cytokine analysis

CAR T cells were cocultured with target (tumor) cells at a 1:1 E:T ratio for 24 h, and supernatant was snap-frozen in liquid nitrogen. Samples were analyzed at Eve Technologies Corporation with a Luminex 200 system (Luminex) using a Human High Sensitivity 14-Plex Discovery Assay (MilliporeSigma) according to the manufacturer’s protocol.

### Retrovirus production and transduction

HEK293T cells (3.5 × 10^6^) were seeded in a 10-cm dish. Medium was replaced with 5 ml of complete DMEM (DMEM supplemented with 10% fetal bovine serum, 2 mM L-glutamine), and cells were transfected with 7.5 μg of pCL-ECO plasmid and 7.5 μg of MSCV plasmid using Lipofectamine LTX with Plus reagent (Invitrogen, 15338030). The transfection mix was prepared in 3 ml of Opti-MEM medium (Gibco, 31985062) and incubated for at least 30 min at room temperature before being added dropwise onto the cell culture. Twenty-four hours after transfection, the medium was exchanged for 6 ml of complete DMEM collection medium. Retrovirus was collected, sterile filtered and frozen at −80 °C for storage at 24 and 48 h.

### Mouse T cell isolation and culture

Spleens from mice were crushed and strained, and T cells were isolated using an EasySep mouse T cell isolation kit (StemCell Technologies, 19851). Cells were cultured in RPMI-1640 (Gibco, 11875093) supplemented with FBS (10%), penicillin–streptomycin (100 U ml^–1^), sodium pyruvate (1 mM), HEPES (10 mM), β-mercaptoethanol (Gibco, 21985-023), MEM non-essential amino acids (1×; Gibco, 11140050) and human IL-2 (200 U ml^–1^; Peprotech, 200-02).

### Mouse CAR T cell generation

The scFv of the 7065 antibody or human CD19 scFv was cloned in the mouse CAR backbone (MSCV plasmid) with N-terminal mouse CD8a signal peptide and C-terminal mouse CD28–mouse CD3z domain, with P2A sequence followed by Thy1.1. Mouse T cells were cultured and activated for 24 h using Dynabeads Mouse T-Expander CD3/CD28 (Gibco, 11452D) and magnetically removed thereafter. T cells (2 × 10^6^) were nucleofected with Cas9 ribonuclease complex (RNP; Lonza, V4SP-3096) using a 4D-Nucleofector 96-well unit (Lonza, AAF-1003S) and Lonza program code DN-100 with 60 pmol of Cas9 protein (QB3 MacroLab) and 120 pmol of sgRNA (UCCCUCCUCUAGAACUUCAC; Synthego) preincubated at 37 °C for 10–15 min. The culture medium was then added to the cells and incubated (37 °C, 5% CO_2_) for 1 h. Retronectin-coated (Takara, T100B) plates were used for culture, and 1 ml of retrovirus carrying the CAR expression cassette was added with 10 μg ml^–1^ polybrene. Cells were spinfected (2,000*g*, 30 °C, 60 min) and incubated overnight in a CO_2_ incubator at 37 °C. Medium was replenished regularly, and cell density was maintained at approximately 2 × 10^6^ cells per ml.

### T cell activation assay

PBMCs were treated with 3 µM ionomycin (Sigma-Aldrich, 407950) + 25 ng ml^–1^ lipopolysaccharide (Sigma-Aldrich, L4391) + 100 U ml^–1^ IL-2 (Prospec, CYT-209) and cultured overnight. Cells were then co-stained with CD3 and CD69 and analyzed by flow cytometry.

### In vitro cytotoxicity assay

AML cell lines were engineered to stably express luciferase using lentiviral transduction. The cell lines were cocultured overnight with CAR T cells. d-Luciferin (150 μg ml^–1^; Gold Biotechnology, LUCK-1G) was added to each well and incubated for 3–5 min at room temperature, followed by luciferase detection using GloMax Explorer (Promega). For each ratio (CAR T cells:tumor cells), the bioluminescence readings from the tumor cells cocultured with untransduced T cells were considered 100% viable for normalization.

### Degranulation assay

CAR T cells were cocultured with tumor cells at ratio of 2:1 for 6 h at 37 °C with anti-CD107a and GolgiStop (BD Biosciences, 51-2092KZ). Cells were washed twice by centrifugation at 500*g* for 5 min at room temperature. Levels of CD107a were then measured with a flow cytometer as a readout of degranulation on GFP^+^ CAR T cells.

### Generation of *ITGB2*-knockout cells

Knockout cell lines or primary T cells were generated using in vitro nucleofection of Cas9 ribonuclease protein complex. Briefly, 2 μl of each sgRNA (100 µM; Synthego Corporation) and recombinant Cas9 protein (40 µM; QB3 MacroLab, University of California, Berkeley) was incubated at 37 °C for 15 min to generate ribonuclease complex, which was then nucleofected using a 4D-Nucleofector (Lonza) with the built-in program DS-137 for cell lines (using Lonza V4XC-2032) and EO-115 for primary T cells (using Lonza V4XP-3032). The sgRNAs used in this study were obtained from the Brunello library^57^ (Supplementary Table 3).

### Off-targeting analysis of sgRNA

Potential off-target cleavage analysis was determined using the online tool CRISPOR^58^. The top five potential hits (genomic loci) were selected for PCR amplification (for primers, see Supplementary Table 2) from genomic DNA of *ITGB2*-knockout and wild-type primary human T cells extracted using a Monarch Genomic DNA Purification kit (New England Biolabs, T3010S). PCR amplicons were sequenced, and data were analyzed using the Synthego ICE Analysis Tool to determine the percentages of insertions and deletions.

### Clonogenic assay

CD34^+^ cells (1 × 10^3^) from healthy donor GM-CSF-mobilized peripheral blood were co-incubated with aITGB2 CAR T cells, My96 CAR T cells, empty CAR T cells, untransduced T cells or medium only (IMDM, 2% FBS and penicillin/streptomycin) at an E:T ratio of 1:1 for 5 h in V-bottom 96-well plates in triplicate in methylcellulose-based medium supplemented with recombinant cytokines (MethoCult H4434 Classic, StemCell Technologies). After 13–14 d, colonies were classified and counted as granulocytes, erythrocytes, monocytes, megakaryocytes; granulocytes, monocytes; granulocytes; monocytes or erythrocytes. Images were acquired with a Keyence microscope using ×10, brightfield and color mode.

### Generation of pathogen-specific T cells

CD45RA^–^ healthy donor PBMCs were isolated using MACS MicroBead Technology (Miltenyi, 130-045-901) and pulsed with a pool of overlapping peptide libraries (15 mers overlapping by 11 amino acids) spanning the entire protein sequences of EBNA1, LMP1 and LMP2; BZLF1 and BRLF1 of Epstein–Barr virus; IE and pp65 of cytomegalovirus; hexon and penton of adenovirus and large T and VP1 or BK virus (JPT technologies) and expanded with IL-7 (10 ng ml^–1^) and IL-15 (5 ng ml^–1^; R&D Systems, 207-IL-010 and BT-015-010). On day 9, the cells received a second stimulation with irradiated pepmix-pulsed autologous ATCs pulsed with the same peptide libraries and irradiated HLA^–^ lymphoblastoid cell lines (ULCLs) at a EBVST:ATC:K562cs/ULCL ratio (EBVST, Epstein-Barr virus-specific T cells; ATCs, activated T cells) of 1:1:5 with IL-7 and IL-15. Expanding cells were split as required and cryopreserved on day 16 of culture. Aliquots were thawed for analysis.

### Generation of HIS mice

The NSG-SGM3 strain (NOD.Cg-*Prkdc*^*scid*^*Il2rg*^*tm1Wjl*^ Tg(CMV-IL3,CSF2,KITLG)1Eav/MloySzJ) obtained from Jackson Laboratories was used to generate HIS mice. All mice used were 6–8 weeks old (either all male or all female for a particular study). Each mouse was treated with busulfan (12.5 mg per kg (body weight)) for 2 d consecutively, followed by 1 d of recovery and injection of 7 × 10^5^ CD34^+^ human hematopoietic cells intravenously through the tail vein. Fully deidentified human CD34^+^-cell enriched blood samples were obtained from the Bone Marrow and Transplantation Laboratory at UCSF and were sorted by magnetic-activated cell sorting using a CD34 MicroBead kit (Miltenyi Biotec, 130-046-702) and incubated with anti-CD3 (Biolegend, 317302, clone OKT3) for T cell depletion 10 min before injection. Flow cytometry for human CD45^+^ cells was performed on mouse blood samples drawn 8 weeks later to determine engraftment efficiency (>1.5% threshold).

### In vivo CAR T cell efficacy in PDX models of AML

All mice used in the experiments were 6–8 weeks old (either all male or all female for a particular study) and were obtained from either Jackson Laboratories (NSG-SMG3) or were bred in-house (NSG) at the Preclinical Therapeutics Core of UCSF. In total, 1 × 10^6^ Nomo1 cells were injected in each mouse. For PDXs, 2 × 10^6^ cells were injected in each mouse irradiated with 250 cGy 4–6 h before injection. In total, 5 × 10^6^ CAR T cells at a 1:1 ratio of CD4^+^:CD8^+^ CAR T cells were injected 5 d after tumor injection. For Nomo1 cells, tumor burden was determined by bioluminescence imaging with a Xenogen In Vivo Imaging System (Caliper Life Sciences). For PDXs, flow cytometry analysis of blood draws and ultrasonography of spleen size were used as readouts for tumor burden. No animals were excluded from the analysis. For such disseminated tumor models, our institution does not specify an allowable maximal tumor burden.

### Statistics and reproducibility

All statistical analyses were performed using GraphPad Prism v.9 unless stated otherwise. The data are represented as mean ± s.e.m., and *P* values of <0.05 were considered statistically significant. No statistical method was used to predetermine sample size, but our sample sizes are similar to those reported in previous publications^11,16,44^. Data distribution was assumed to be normal, but this was not formally tested. No data were excluded from the analyses. Animals were randomized based on body weight before treatment. The Preclinical Core Facility staff was blinded to mouse treatment and relevant outcomes. The other investigators were not blinded to allocation during the experiments and outcome assessment.

### Reporting summary

Further information on research design is available in the Nature Portfolio Reporting Summary linked to this article.

### Supplementary information

Supplementary Information Supplementary Information 1 and 2.

Reporting Summary

Supplementary Table Supplementary Tables 1–3.

### Source data

Source Data Statistical source data for all figures in separate tabs of a single Excel sheet.

## Data Availability

Raw proteomic data generated here have been deposited at the ProteomeXchange/PRIDE repository under accession numbers PXD035404, PXD035589 and PXD035591. Previously published bulk RNA-seq datasets (TCGA AML, BEAT AML and TARGET AML) that are reported here are available under accession codes phs000178, phs001657 and phs000218, respectively, through the NIH/NCI GDC Data Portal. The following are the other public database repertories used in the study: https://www.proteinatlas.org/ (ITGB2, CD33, CD123 and CLEC12A), https://www.proteinatlas.org/humanproteome/single+cell+type, https://www.proteinatlas.org/humanproteome/single+cell+type/blood+&+immune+cells and https://www.rcsb.org/ (PDB 5E6R). Source data are provided with this paper.

## Code availability

The Ving software package for analysis of DSSO XL–MS data is available at GitHub at https://github.com/mhoopmann/Ving.
