# Supplementary material for: Structural surfaceomics reveals an AML-specific conformation of integrin β2 as a CAR T cellular therapy target
Source: Nat Cancer. 2023 Oct 30;4(11):1592–609. doi: 10.1038/s43018-023-00652-6 (PMC10663162; doi:10.1038/s43018-023-00652-6)
Supplement: Supplementary file 1 — Supplementary Information 1 and 2. [file 43018_2023_652_MOESM1_ESM.pdf]

nature cancer

Article https://doi.org/10.1038/s43018-023-00652-6

# Structural surfaceomics reveals an AML-specific conformation of integrin $\beta_2$ as a CAR T cellular therapy target

In the format provided by the authors and unedited

## Supplementary information- 1

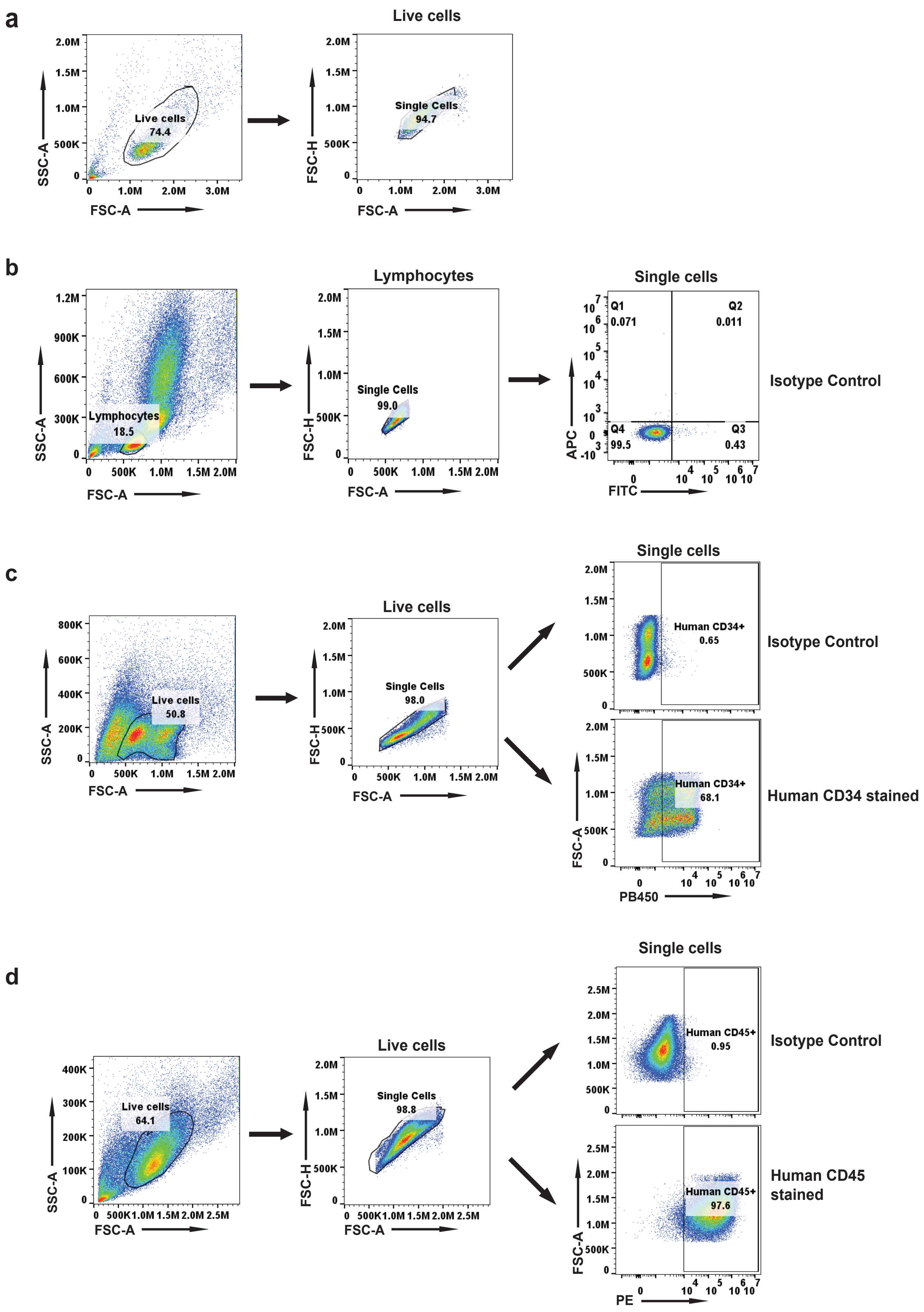


**Supplementary information- 1 -** Flow cytometry Gating stategies

## Supplementary information- 2

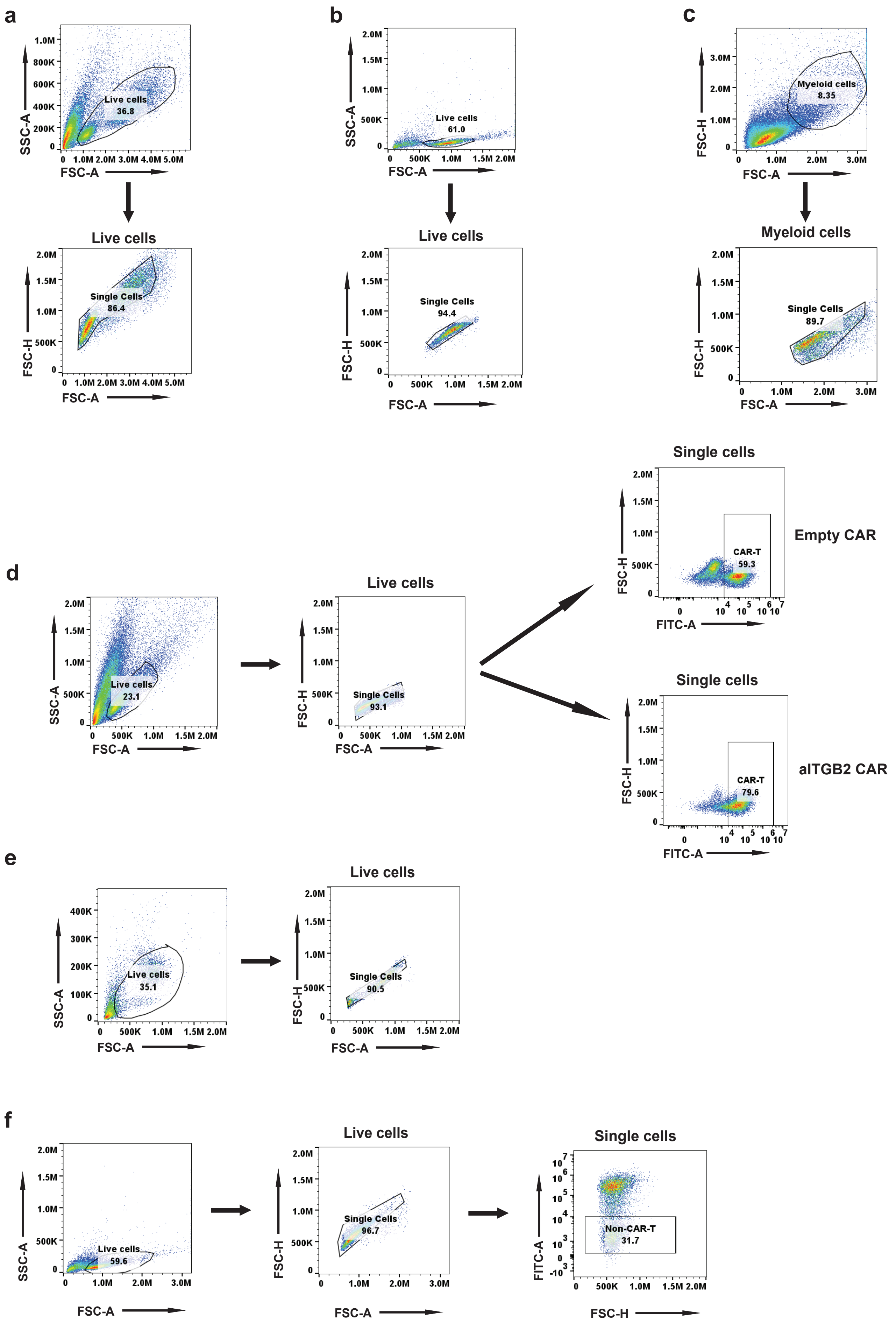


**Supplementary information- 2 -** Flow cytometry Gating stategies
