# Supplementary material for: Structural surfaceomics reveals an AML-specific conformation of integrin β2 as a CAR T cellular therapy target
Source: Nat Cancer. 2023 Oct 30;4(11):1592–609. doi: 10.1038/s43018-023-00652-6 (PMC10663162; doi:10.1038/s43018-023-00652-6)
Supplement: Supplementary file 2 — Reporting Summary [file 43018_2023_652_MOESM2_ESM.pdf]

nature portfolio

Corresponding author(s): Wiita, Arun P.

Last updated by author(s): Jul 15, 2023

Reporting Summary

Nature Portfolio wishes to improve the reproducibility of the work that we publish. This form provides structure for consistency and transparency in reporting. For further information on Nature Portfolio policies, see our Editorial Policies and the Editorial Policy Checklist.

Statistics

For all statistical analyses, confirm that the following items are present in the figure legend, table legend, main text, or Methods section.

n/a	Confirmed	
☐	☒	The exact sample size (*n*) for each experimental group/condition, given as a discrete number and unit of measurement
☐	☒	A statement on whether measurements were taken from distinct samples or whether the same sample was measured repeatedly
☐	☒	The statistical test(s) used AND whether they are one- or two-sided<br>*Only common tests should be described solely by name; describe more complex techniques in the Methods section.*
☒	☐	A description of all covariates tested
☐	☒	A description of any assumptions or corrections, such as tests of normality and adjustment for multiple comparisons
☐	☒	A full description of the statistical parameters including central tendency (e.g. means) or other basic estimates (e.g. regression coefficient) AND variation (e.g. standard deviation) or associated estimates of uncertainty (e.g. confidence intervals)
☐	☒	For null hypothesis testing, the test statistic (e.g. *F*, *t*, *r*) with confidence intervals, effect sizes, degrees of freedom and *P* value noted<br>*Give P values as exact values whenever suitable.*
☒	☐	For Bayesian analysis, information on the choice of priors and Markov chain Monte Carlo settings
☒	☐	For hierarchical and complex designs, identification of the appropriate level for tests and full reporting of outcomes
☒	☐	Estimates of effect sizes (e.g. Cohen's *d*, Pearson's *r*), indicating how they were calculated

Our web collection on statistics for biologists contains articles on many of the points above.

Software and code

Policy information about availability of computer code

Data collection	CytExpert v2.4.0.28, Thermo Xcalibur 4.0.27.19, Compass Hystar 6.2, ForteBio's Octet Systems software v9.0, NanoDrop 2000c software v1.6, Living Image, version 4.7.4, Image Lab v2.4.0.03, Agilent OpenLab CDS ChemStation v2.3, Glomax Explorer - Promega version is 3.2.3
Data analysis	Flow cytometry: FlowJo v. 10.8.1; Statistics: GraphPad Prism 9; Mass spectrometry: FragPipe v14.0, pLink-2, Protein Prospector v.6.3.5, TPP 5.2.0. , SnapGene 5.2; Custom code of Ving - https://github.com/mhoopmann/Ving

For manuscripts utilizing custom algorithms or software that are central to the research but not yet described in published literature, software must be made available to editors and reviewers. We strongly encourage code deposition in a community repository (e.g. GitHub). See the Nature Portfolio guidelines for submitting code & software for further information.

Data

Policy information about availability of data

All manuscripts must include a data availability statement. This statement should provide the following information, where applicable:

- Accession codes, unique identifiers, or web links for publicly available datasets
- A description of any restrictions on data availability
- For clinical datasets or third party data, please ensure that the statement adheres to our policy

Proteomic data generated in this study was deposited to ProteomeXchange via the PRIDE database with accession number - PXD035404, PXD035589 and PXD035591. There are no restrictions on data availability. Other public database repositories used in the study are as follows:

https://www.proteinatlas.org/ (ITGB2, CD33, CD123, CLEC12A) ; https://portal.gdc.cancer.gov/ (TCGA, BEAT, TARGET); https://www.rcsb.org/ (PDB: 5E6R)
https://portal.gdc.cancer.gov/

Research involving human participants, their data, or biological material

Policy information about studies with human participants or human data. See also policy information about sex, gender (identity/presentation), and sexual orientation and race, ethnicity and racism.

Reporting on sex and gender	The patient samples used in this study were de-identified.
Reporting on race, ethnicity, or other socially relevant groupings	The patient samples used in this study were de-identified.
Population characteristics	The patient samples used in this study were de-identified.
Recruitment	All the patient samples used in the study were obtained from the UCSF Hematologic Malignancies Tissue Bank and the Pediatric Hematopoietic Tissue Cell Bank.
Ethics oversight	All the patient samples used in the study were obtained under IRB-approved protocols by the UCSF Committee on Human Research and following the Declaration of Helsinki.

Note that full information on the approval of the study protocol must also be provided in the manuscript.

Field-specific reporting

Please select the one below that is the best fit for your research. If you are not sure, read the appropriate sections before making your selection.

☒ Life sciences ☐ Behavioural & social sciences ☐ Ecological, evolutionary & environmental sciences

For a reference copy of the document with all sections, see nature.com/documents/nr-reporting-summary-flat.pdf

Life sciences study design

All studies must disclose on these points even when the disclosure is negative.

Sample size	All the quantitative experiments were performed with n ≥ 3. The others were also performed with multiple technical and biological replicates as stated in the legends. For primary sample analysis there was no pre-determined sample size and no specific power analysis to determine the number of primary samples to be used. Sample sizes were chosen based on standards in the field for similar experiments. Statistical significance of outcomes was determined as described.
Data exclusions	No data exclusions.
Replication	Multiple biological and/or technical replicates were performed for all experiments unless stated otherwise, and are specifically noted in the figure legends. We did not experience any inability to reproduce results.
Randomization	Animals used in the study were randomized before CAR-T treatment. For in vitro and proteomic experiments as described randomization is not applicable to the reported design
Blinding	Preclinical Core Facility staffs were blinded to murine treatment and relevant outcomes. For other in vitro studies blinding was not possible due to sample preparation and execution by a single experimenter.

Reporting for specific materials, systems and methods

We require information from authors about some types of materials, experimental systems and methods used in many studies. Here, indicate whether each material, system or method listed is relevant to your study. If you are not sure if a list item applies to your research, read the appropriate section before selecting a response.

Materials & experimental systems

n/a	Involved in the study
☐	☒ Antibodies
☐	☒ Eukaryotic cell lines
☒	☐ Palaeontology and archaeology
☐	☒ Animals and other organisms
☒	☐ Clinical data
☒	☐ Dual use research of concern
☒	☐ Plants

Methods

n/a	Involved in the study
☒	☐ ChIP-seq
☐	☒ Flow cytometry
☒	☐ MRI-based neuroimaging

Antibodies

Antibodies used

The antibodies used in this study are CD3 (Biolegend, 980008, 300412, clone- UCHT1, Lot no.- B341478, B326668), CD19 (Biolegend, 363006, 363036, clone- SJ25C1, Lot no.- B342061, B289109), CD45 (Biolegend, 368512, clone- 2D1, Lot no.- B352919), CD14 (Biolegend, 367118, 367104, clone- 63D3, Lot no.- B356227, B274117), CD34 (Biolegend, 343510, clone- 581, Lot no.- B351598), CD69 (Biolegend, 985206, clone- FN50, Lot no.- B352653), CD11a/CD18 (Biolegend, 363406, 363416, clone- m24, Lot no.- B344166, B283734), CD18 (Biolegend, 302106, clone- TS1/18, Lot no.- B272927), CD33 (Biolegend, 303404, clone- WM53, Lot no.- B349851), CD62L (BD Biosciences, 559772, clone: DREG-56, Lot no.- 2031767), CD45RA (Thermo Fisher Scientific, 12-0458-42, clone: HI100, Lot no.- 2460218), CD16 (Biolegend, 302032, clone- 3G8, Lot no.- B346619), CD25 (Invitrogen, 17-00259-42, clone- BC96, Lot no.- 2382945), LAG3 (Invitrogen, 48-2239-42, clone- 3DS223H, Lot no.- 2547905), TIM3 (Biolegend, 345005, clone- F38-2E2, Lot no.- B354359), PD1 (Biolegend, 329908, clone- EH12.2H7, Lot no.- B362224), CD45RO (BD Biosciences, 555493, clone- UCHL1, Lot no.- 328710), CCR7 (Biolegend, 353214, clone- G043H7, Lot no.- B286358) and CD64 (Biolegend, 305018, clone- 10.1, Lot no.- B272932). All the respective isotype/secondary antibodies used were procured and used as per the vendor's instructions.

Validation

CD3 (Biolegend, 980008, 300412, clone- UCHT1); Validated by staining T cells selectively expanded from PBMC using T cell media and stimulating beads. Also, validated with isotypes staining as a background. References: (1) Barclay N, et al. 1993. The Leucocyte FactsBook. Academic Press. San Diego. (2) Beverly P, et al. 1981. Eur. J. Immunol. 11:329. (3) Lanier L, et al. 1986. J. Immunol. 137:2501-2507.
CD19 (Biolegend, 363006, 363036, clone- SJ25C1); Validated using CRISPR-Cas9 knockout B cell line and also with isotypes staining as a background. Also, validated with cellular toxicity assay (Nix MA, et al. 2021. Cancer Discovery) where selectively CD19 expressing cells were depleted, with appropriate biological controls. References: (1) Liu C, et al. 2021. Cell. 184(7):1836-1857.e22. (2) Rodda LB, et al. 2020. Cell. 184(1):169-183.e17. (3) Mori A, et al. 2021. Cells. :10.

CD45 (Biolegend, 368512, clone- 2D1); Validated with detection of human cells in mice injected with human cells and not binding to mice cells. Also, validated with isotypes staining as a background. References: (1) Zhou Y, et al. 2020. Cancer Cell. 38(6):818-828.e5. (2) Boyd DF, et al. 2020. Nature. 587:466. (3) Azizi E et al. 2018. Cell. 174(5):1293-1308 e36.

CD14 (Biolegend, 367118, 367104, clone- 63D3); Validated with isotypes staining as a background and with cellular cytotoxicity assay where myeloid cells expressing CD14 were selectively depleted, with appropriate biological controls. References: (1) Mehta AK, et al. 2021. Nat Cancer. 2:66. (2) James KR, et al. 2020. Nat Immunol. 1.113194444. (3) Wang T et al. 2018. Immunity. 49(3):504-514

CD34 (Biolegend, 343510, 343510, clone- 581); Validated staining MACS sorted CD34+ cells and later injected in mice to generate Human Immune System mice which reconstituted human immune system. Also, validated with isotypes staining as a background. References: (1) Kohn L, et al. 2012. Nat Immunol. 13:963. (2) Chabi S, et al. 2020. Cell Reports. 29(8):2307-2320.e6. (3) Sharma R, et al. 2021. Nat Commun. 12:472.

CD69 (Biolegend, 985206, clone- FN50); Validated with artificial activation of T cell using ionomycin and also using isotypes staining as a background. References: (1) Schlossman S, et al. 1995. Oxford University Press. (2) Testi R, et al. 1994. Immunol Today. 15:479-83.

CD11a/CD18 (Biolegend, 363406, 363416, clone- m24); Validated with artificial activation of the antigen (active Itgβ2) using Mn2+ ions and also with isotype staining as a background. References: (1) Karampatzakis A, et al. 2021. Front Immunol. 12:641521. (2) Hogg, N. & Selvendran, Y. et al. 1985. Cell Immunol 92, 247–253. (3) Dransfield I, et al. 1992. Journal of Cell Biology 116, 219–226.

CD18 (Biolegend, 302106, clone- TS1/18); Validated using CRISPR-Cas9 knockout of T cells and also with isotypes staining as a background. References: (1) Buffone A, et al. 2018. J Cell Sci. 131: (2) Lutter L, et al. 2021. Cell Mol Gastroenterol Hepatol. 12:1567. (3) Kao TI, et al. 2021. Br J Pharmacol. 178:4069.

CD33 (Biolegend, 303404, clone- WM53); Validated with isotypes staining as a background, with successful staining of several myeloid cell lines, CD33 being a myeloid marker . References: (1) Tcheng M, et al. 2021. Blood. 137:3518. (2) Subramaniam A, et al. 2020. Blood. 136:2151. (3) Schmiderer L, et al. 2020. Proc Natl Acad Sci U S A. 117:21267.

CD62L (BD Biosciences, 559772, clone: DREG-56); Validated with isotypes staining as a background. References: (1) Kishimoto TK, et al. 1990. Proc Natl Acad Sci U S A. 87(6):2244-2248. (2) Kishimoto TK, et al. 1991. Blood. 78(3):805-811 (3) Schlossman SF, et al. 1993. Oxford: Oxford University Press

CD45RA (Thermo Fisher Scientific, 12-0458-42, clone: HI100); Validated with isotypes staining as a background. References: (1) Psaila B, et al. 2020. Mol Cell 78(3):477-492 (2) Bell CC, et al. 2019. Nat Commun. 10(1):2723 (3) Eyquem J, et al. 2017. Nature. 543(7643):113-117

CD16 (Biolegend, 302032, clone- 3G8);
Validated with isotypes staining as a background and with cellular cytotoxicity assay where myeloid cells expressing CD16 were selectively depleted, with appropriate biological controls. References: (1) Tiwari-Heckler S, et al. 2021. Cell Rep. 37:109897. (2) Hegewisch-Solloa E, et al. 2021. J Immunol. 207:950 (3) Rhoades NS, et al. 2022. Cell Rep. 39:110725.

CD25 (Invitrogen, 17-00259-42, clone- BC96); Validated with isotypes staining as a background. References: (1) Vallejo-Gracia A, et al. 2020. Nature Microbiology. (9):1144-1157. (2) Duscha A, et al. 2020. Cell. 180(6):1067-1080 (3) Barry KC, et al. 2018. Nature Medicine. (8):1178-1191

LAG3 (Invitrogen, 48-2239-42, clone- 3DS223H); Validated with isotypes staining as a background. References: (1) Miles B, et al. 2015. Nature Communications. 6:8608 (2) Belkina AC, et al. 2017. Cytometry A. (2):175-179 (3) Stunnenberg M, et al. 2020. Viruses. 12(7):764

TIM3 (Biolegend, 345005, clone- F38-2E2); Validated with isotypes staining as a background. References: (1) Jung IY, et al. 2022. Sci Transl Med. 14:eabn7336. (2) Wei F, et al. 2013. Proc Natl Acad Sci U S A. 110:2480. (3) de Boer B et al. 2018. Cancer cell. 34(4):674-689 .

PD1 (Biolegend, 329908, clone- EH12.2H7); Validated with isotypes staining as a background. References: (1) Cao B, et al. 2022. Nat Commun. 13:6203. (2) Montes de Oca M, et al. 2016. Cell Rep. 17:399-412. (3) Wang Z, et al. 2018. Nat Commun. 9:824.

CD45RO (Biolegend, 304206, clone- UCHL1); Validated with isotypes staining as a background. References: (1) Argüello RJ, et al. 2020. Cell Metab. 32:1063. (2) Carisey AF, et al. 2018. Curr Biol. 28:489. (3) Guo J, et al. 2022. Front Cell Dev Biol. 9:775599.

CCR7 (Biolegend, 353214, clone- G043H7); Validated with isotypes staining as a background. References: (1) Li M, et al. 2021. J Clin Invest. 131: (2) Miao L, et al. 2021. Clin Transl Med. 11:e395. (3) Fajgenbaum DC, et al. 2019. J Clin Invest. 130:4451.

CD64 (Biolegend, 305018, clone- 10.1); Validated with isotypes staining as a background. References: (1) Magg T, et al. 2021. Sci Immunol. 6:. (2) Holl V, et al. 2004. J. Immunol. 173:6274. (3) Bruhns P, et al. 2008. Blood 113:3716.

Eukaryotic cell lines

Policy information about cell lines and Sex and Gender in Research

Cell line source(s)	Nomo-1 (Cat. No. ACC 542) and BV-173 (Cat. No. ACC 20) were originally obtained from DSMZ. THP1 (Cat. No. TIB 202), HL60 (Cat. No. CCL 240), MV411 (Cat. No. CRL 9591), Jurkat (Cat. No. TIB 152), U937 (Cat. No. CRL-1593.2), HEK-293T (Cat. No. CRL-3216), Namalwa (Cat. No. CRL-1432) and S49.1 (Cat. No. TIB 28) were obtained from ATCC.
Authentication	Cell lines were authenticated using STR genotyping.
Mycoplasma contamination	Cell lines tested negative for mycoplasma contamination in routine testing.
Commonly misidentified lines (See ICLAC register)	HL60 and U-937 used in this paper was confirmed with STR genotyping on 2/17/23 and 7/21/23, respectively.

Animals and other research organisms

Policy information about studies involving animals; ARRIVE guidelines recommended for reporting animal research, and Sex and Gender in Research

Laboratory animals	NSG and NSG-SGM3 mice used and were either all male or all female in specific study as stated in the legends. Age of the animls used were 6-8 weeks.
Wild animals	No wild animals were used in this study.
Reporting on sex	Sex considerations were beyond the scope of the study design. Thus, sex was not considered in study design. For a particular animal study either all females or all males were used as stated in the method section.
Field-collected samples	The study did not involve any field-collected samples.
Ethics oversight	All murine experiments were conducted in accordance with an approved protocol by the UCSF Institutional Animal Care and Usage Committee

Note that full information on the approval of the study protocol must also be provided in the manuscript.

Flow Cytometry

Plots

Confirm that:

☒ The axis labels state the marker and fluorochrome used (e.g. CD4-FITC).

☒ The axis scales are clearly visible. Include numbers along axes only for bottom left plot of group (a 'group' is an analysis of identical markers).

☒ All plots are contour plots with outliers or pseudocolor plots.

☒ A numerical value for number of cells or percentage (with statistics) is provided.

Methodology

Sample preparation	Immunostaining of cells were performed as per the instructions from antibody vendor unless stated otherwise. Briefly, 1 million cells were resuspended in 100 µl of FACS buffer (PBS + 2% FBS) with 1 ug antibody added to it. The cells were incubated at 4C for 10-15 minutes and then washed thrice with the FACS buffer. For staining active form of ITGB2, antibody incubation step was performed at 37C for 1 hour. In case of staining primary AML cells for activated ITGB2, recipe of FACS buffer was RPMI-1640 + 5% FBS + 2% BSA + 50 µg/ml DNase-I (Gold Biotechnology, D-301-500). For all other primary cell staining, FACS buffer recipe was D-PBS + 5% FBS + 2% BSA + 5 mM EDTA + 50 µg/ml DNase-I with Human Trustain (Biolegend, 422302). All the compensation was done using UltraComp eBeads™ Compensation Beads (Invitrogen, 01-2222-42). All the flow cytometry analysis was done with Cytoflex (Beckman Coulter) and data was analyzed using FlowJo_v10.8.1. The antibodies used in this study are CD3 (Biolegend, 980008, 300412, clone- UCHT1), CD19 (Biolegend, 363006, 363036, clone-SJ25C1) , CD45 (Biolegend, 368512, clone- 2D1), CD14 (Biolegend, 367118, 367104, clone- 63D3), CD34 (Biolegend, 343510, 343510, clone- 581), CD69 (Biolegend, 985206, clone- FN50), CD11a/CD18 (Biolegend, 363406, 363416, clone- m24), CD18 (Biolegend, 302106, clone- TS1/18), CD33 (Biolegend, 303404, clone- WM53), CD62L (BD Biosciences, 559772, clone: DREG-56), CD45RA (Thermo Fisher Scientific, 12-0458-42, clone: HI100), CD16 (Biolegend, 302032, clone- 3G8), CD25 (Invitrogen, 17-00259-42, clone- BC96), LAG3 (Invitrogen, 48-2239-42, clone- 3DS223H), TIM3 (Biolegend, 345005, clone-F38-2E2), PD1 (Biolegend, 329908, clone- EH12.2H7), CD45RO (Biolegend, 555493, clone- UCHL1), CCR7 (Biolegend, 353214, clone- G043H7) and CD64 (Biolegend, 305018, clone- 10.1). Secondary antibody used was anti-human IgG Fc antibody (Biolegend, 410720). All the respective isotype antibodies used were procured and used as per the vendor's instructions.
Instrument	BC Cytoflex was used for all analytical flow cytometry.
Software	FlowJo versions 10.8.1 were used.
Cell population abundance	For cell line analysis, cell population was gated on all live, singlet cells. These cells were quantified for abundance of the population of interest.
Gating strategy	All the gating strategies have been shown in the extended data and are described in the legends.

☒ Tick this box to confirm that a figure exemplifying the gating strategy is provided in the Supplementary Information.
